# Conversion from off-pump to on-pump coronary artery bypass grafting: impact of surgeon and anaesthetist experience

**DOI:** 10.1093/icvts/ivad205

**Published:** 2023-12-20

**Authors:** Vasileios Ntinopoulos, Achim Haeussler, Dragan Odavic, Nestoras Papadopoulos, Laura Rings, Stak Dushaj, Hector Rodriguez Cetina Biefer, Omer Dzemali

**Affiliations:** Department of Cardiac Surgery, University Hospital of Zurich, Zurich, Switzerland; Department of Cardiac Surgery, Municipal Hospital of Zurich—Triemli, Zurich, Switzerland; Department of Cardiac Surgery, University Hospital of Zurich, Zurich, Switzerland; Department of Cardiac Surgery, Municipal Hospital of Zurich—Triemli, Zurich, Switzerland; Department of Cardiac Surgery, University Hospital of Zurich, Zurich, Switzerland; Department of Cardiac Surgery, Municipal Hospital of Zurich—Triemli, Zurich, Switzerland; Department of Cardiac Surgery, University Hospital of Zurich, Zurich, Switzerland; Department of Cardiac Surgery, Municipal Hospital of Zurich—Triemli, Zurich, Switzerland; Department of Cardiac Surgery, University Hospital of Zurich, Zurich, Switzerland; Department of Cardiac Surgery, Municipal Hospital of Zurich—Triemli, Zurich, Switzerland; Department of Cardiac Surgery, University Hospital of Zurich, Zurich, Switzerland; Department of Cardiac Surgery, Municipal Hospital of Zurich—Triemli, Zurich, Switzerland; Department of Cardiac Surgery, University Hospital of Zurich, Zurich, Switzerland; Department of Cardiac Surgery, Municipal Hospital of Zurich—Triemli, Zurich, Switzerland; Center for Translational and Experimental Cardiology (CTEC), Department of Cardiology, University Hospital of Zurich, University of Zurich, Zurich, Switzerland; Department of Cardiac Surgery, University Hospital of Zurich, Zurich, Switzerland; Department of Cardiac Surgery, Municipal Hospital of Zurich—Triemli, Zurich, Switzerland; Center for Translational and Experimental Cardiology (CTEC), Department of Cardiology, University Hospital of Zurich, University of Zurich, Zurich, Switzerland

**Keywords:** Off-pump coronary artery bypass grafting, Off-pump coronary artery bypass grafting, OPCAB, Conversion, Surgeon experience, Anaesthetist experience

## Abstract

**OBJECTIVES:**

Intraoperative conversion from off-pump to on-pump coronary artery bypass grafting (CABG) is associated with increased postoperative morbidity and mortality. The aim of this study is to assess the impact of surgeon and anaesthetist experience on the conversion rate.

**METHODS:**

We performed a retrospective analysis of the data of all patients who underwent planned off-pump CABG in a single centre in 2007–2021, some of whom were non-electively converted to on-pump. Surgeon and anaesthetist experience were assessed by the number of off-pump bypass procedures per year. Multivariable logistic regression analysis was used to assess the impact of surgeon and anaesthetist experience on conversion rate.

**RESULTS:**

A total of 2742 patients met the inclusion criteria. Ninety-four (3.4%) patients underwent non-elective conversion to on-pump surgery. Converted patients had significantly higher mortality [11 (11.7%) vs 35 (1.3%), *P* < 0.0001] in comparison to non-converted patients. Anaesthetist experience was found to be a risk factor for conversion (*P* = 0.011). Surgeon experience did not significantly affect conversion rate (*P* = 0.51). Other risk factors for conversion were female gender [odds ratio 2.65 (95% confidence interval 1.65–4.26), *P* = 0.0001] and left ventricular ejection fraction ≤35% [odds ratio 1.91 (95% confidence interval 1.05–3.49), *P* = 0.040].

**CONCLUSIONS:**

Conversion from off-pump to on-pump CABG is associated with worse postoperative outcomes. Limited experience of anaesthetists in off-pump bypass surgery is associated with a higher conversion rate.

**Figure:**
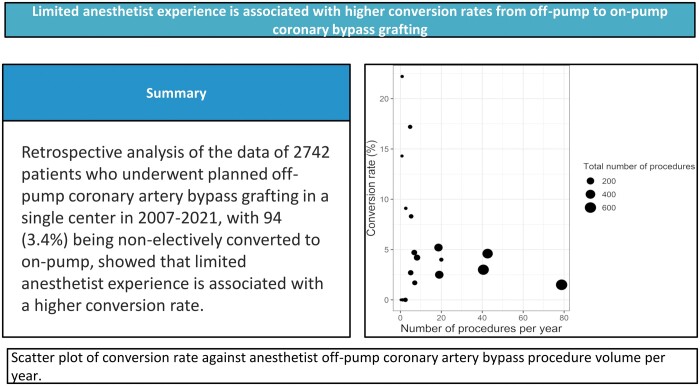

## INTRODUCTION

Coronary artery bypass grafting (CABG) is a safe and effective therapy for the treatment of patients with coronary artery disease [1–3]. Off-pump CABG offers several advantages over conventional on-pump CABG and has been shown to reduce morbidity and mortality in selected patient subgroups such as octogenarians and patients with contraindications for cannulation or manipulation of the aorta compared to on-pump CABG [4–7]. However, intraoperative non-elective conversion from off-pump to on-pump CABG may occur in 1.4–16% of off-pump CABG procedures and is a complication leading to an increase in postoperative morbidity and mortality [8–13]. Published studies have identified various risk factors for conversion from off-pump to on-pump CABG, such as surgeon experience, hospital case volume, advanced patient age, female gender, low left ventricular ejection fraction (LVEF), non-elective surgery and recent myocardial infarction [9, 13–16]. Even though off-pump CABG surgery is a team effort requiring experience and alertness of both surgeon and anaesthetist in order to early detect severe haemodynamic instability and avoid non-elective conversion to on-pump CABG, no published studies have assessed the effect of anaesthetist experience on the rate of conversion from off-pump to on-pump CABG.

Aim of this study is to assess the impact of surgeon and anaesthetist experience on the rate of conversion from off-pump to on-pump CABG, and to examine the effect of conversion on postoperative outcomes.

## PATIENTS AND METHODS

### Ethical statement

The study was approved, and individual informed consent was waived by the local ethics committee (Business Administration System for Ethics Committees/BASEC-Number 2021-02461).

### Study design

We performed a retrospective analysis of the data of all patients who underwent planned off-pump CABG in a single centre in 2007–2021, some of whom were non-electively converted to on-pump. Patients who underwent conversion from off-pump to on-pump CABG due to haemodynamic instability before sternotomy, during internal thoracic artery harvesting or directly before or after opening the pericardium, and patients who had elective conversion before any manipulation to the heart were excluded from the analysis. Additionally, patients with previous myocardial infarction in the last 24 h before surgery, or previous cardiac surgery, and cases with no data about surgeon or anaesthetist procedure volume per year were also excluded. Almost all patients who underwent CABG in our cardiac surgery department during the study period were primarily operated on with the off-pump technique. An interdisciplinary discussion with the cardiologists preceded every decision to perform the procedures. A heart positioner and/or a tissue stabilizer were used as stabilizing devices. Deep pericardial stitches and/or gauze pads were used in cases of insufficient exposure of the coronary anastomosis site. All procedures were performed with the use of an intraluminal coronary shunt during each coronary anastomosis. A carbon dioxide blower was used for better visualization of the anastomosis site during surgery. No particular precautions regarding the partial blood pressure of CO_2_ were implemented during off-pump coronary artery bypass grafting (OPCAB), targeting normocapnia during the procedure. Even though the anaesthesiologists performing anaesthesia and assisting these procedures were board certified anaesthetists with various levels of previous experience in cardiac anaesthesiology, there has yet to be an official cardiac anaesthesiology qualification in our country, therefore, no formal national-level evaluation of the qualification level of each anaesthesiologist on cardiac anaesthesia was available.

The following patient data were collected: preoperative [age, gender, body mass index, Canadian Cardiovascular Society class, New York Heart Association class, arterial hypertension, dyslipidaemia, diabetes mellitus, smoking status, chronic obstructive pulmonary disease, previous cerebrovascular insult, peripheral arterial disease, LVEF (no data regarding differentiation between acute and chronic heart failure available), blood creatinine level, previous percutaneous coronary intervention, ≥50% stenosis of the left main coronary artery, beta-blocker intake preoperatively, non-elective surgery and additive European system for cardiac operative risk evaluation (EuroSCORE)], intraoperative (surgeon, anaesthetist) and in-hospital postoperative (cardiopulmonary resuscitation, installation of intra-aortic balloon pump or extracorporeal membrane oxygenation, renal replacement therapy, re-exploration for bleeding or cardiac tamponade, atrial fibrillation, blood product transfusion, intubation duration, intensive care unit stay, postoperative hospital stay and mortality). Postoperative outcomes were compared between the converted and non-converted patients. Multivariable logistic regression analysis was used to assess the impact of surgeon and anaesthetist experience on conversion rate. Surgeon and anaesthetist experience were assessed by the number of off-pump CABG procedures per year.

### Data analysis

The statistical analyses were performed with IBM SPSS Statistics for Windows, Version 27.0 (IBM, Armonk, NY, USA) and R, Version 4.3.1 (R Foundation for Statistical Computing, Vienna, Austria). Categorical variables are presented as counts (percentages), and continuous variables as mean (standard deviation) by normally distributed data, and median (first and third quartile) by non-normally distributed data. The normality of data distribution was assessed using mainly *Q*–*Q* plot and histogram inspection and secondarily with the Kolmogorov–Smirnov and the Shapiro–Wilk test. Continuous data were compared with the Student’s *t*-test or the Mann–Whitney *U*-test according to the normality of data distribution. Categorical data were compared with the chi-squared or Fisher’s exact test according to the number of cells with an expected count of <5 in the respective contingency tables. Univariable and multivariable logistic regression was performed to assess for factors affecting conversion from off-pump to on-pump CABG. The effect of surgeon and anaesthetist off-pump CABG procedure volume per year on the conversion rate was assessed with univariable and multivariable logistic regression. Restricted cubic spline transformation of the surgeon and anaesthetist off-pump CABG procedure volume per year, with placement of 3 knots in the 10th, 50th and 90th quantile of each variable were performed using the ‘rcs’ function of the ‘rms’ package in R. These transformations were then used for the univariable and multivariable logistic regressions. Multivariable logistic regression included surgeon and anaesthetist procedure volume per year, age ≥65 years, female gender, LVEF ≤35%, non-elective surgery and left main coronary artery stenosis (≥50%). Multicollinearity assessment for the predictors of the multivariable model was performed with the calculation of the variance inflation factor (VIF), using the ‘vif’ function of the ‘car’ package in R. In order to monitor and detect changes in conversion rate during the study period for each surgeon and anaesthetist, cumulative sum (CUSUM) control charts were constructed with the ‘cusum’ package in R. For the calculation of the control limit of the CUSUM, the total cohort size (*n* = 2742) was used as sample size and the mean conversion-rate of the entire cohort (3.4%) as accepted failure probability. All tests were two-sided, and the level of statistical significance was set at 0.05.

## RESULTS

**Figure 1:**
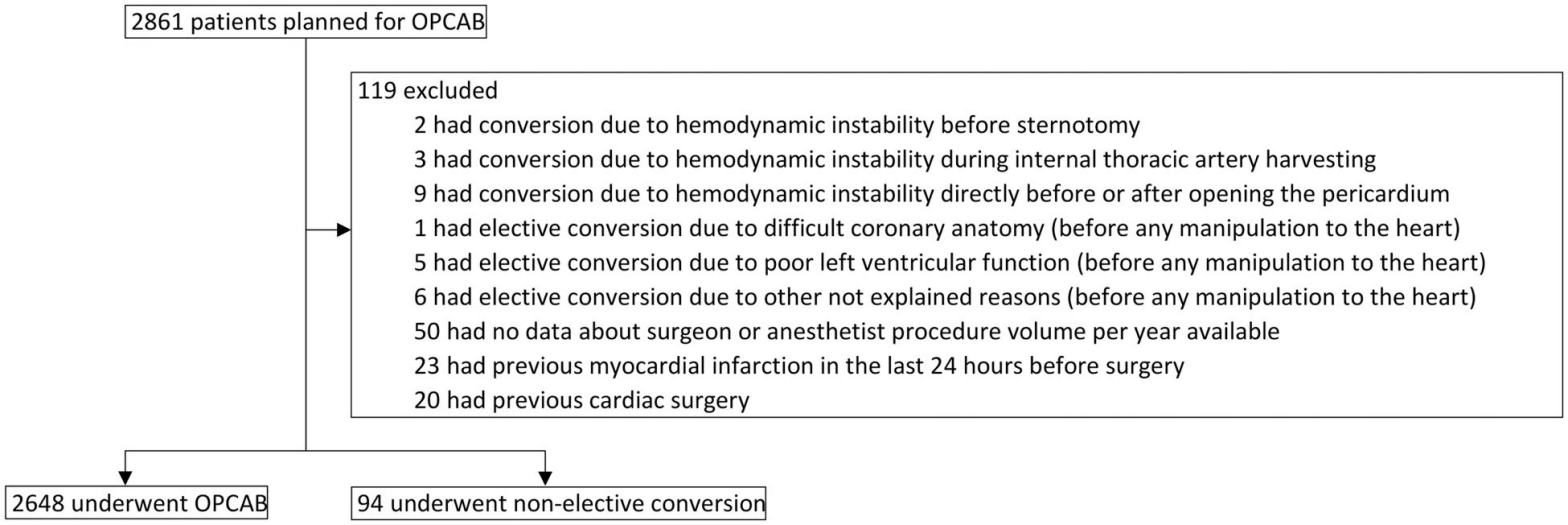
Flow chart of included and excluded patients.

A total of 2742 patients met the inclusion criteria. Figure 1 depicts the exact number of patients included and excluded from the analysis. Ninety-four (3.4%) patients underwent non-elective conversion to on-pump surgery. Of these, 88 were performed on-pump without cardioplegia and 6 on-pump with cardioplegia. The 15-year study period was divided into 3 subperiods (first 2007–2011, second 2012–2016, third 2017–2021) and the number of conversions was 9 (1%) vs 32 (3.2%) vs 53 (6.3%) for each period respectively (*P* < 0.0001). There was no statistically significant difference in mortality between the 3 subperiods [13 (1.5%) vs 22 (2.2%) vs 11 (1.3%), *P* = 0.31]. Median additive EuroSCORE was statistically significantly different between the 3 subperiods [6 (3–8) vs 4 (2–7) vs 4 (2–6), *P* < 0.0001].

The postoperative in-hospital outcomes of the study population are presented in Table 1. Patients non-electively converted from off-pump to on-pump CABG had a statistically significantly higher rate of renal replacement therapy [11 (12.6%) vs 48 (1.8%), *P* < 0.0001], re-exploration for bleeding or cardiac tamponade [12 (14.5%) vs 63 (2.5%), *P* < 0.0001], transfusion of red cell concentrates [1 (0–4.5) vs 0 (0–1) units, *P* < 0.0001], longer intubation duration [6 (4–21) vs 5 (4–8) hours, *P* = 0.0006], longer postoperative hospital stay [11 (8–14) vs 9 (7–11) days, *P* = 0.0001] and higher mortality [11 (11.7%) vs 35 (1.3%), *P* < 0.0001] in comparison to non-converted patients.

**Table 1:** Postoperative in-hospital outcomes of the study population

	Off-pump CABG (*n* = 2648)	Conversion to on-pump CABG (*n* = 94)	*P*-Value
Cardiopulmonary resuscitation	34 (1.3)	6 (6.9)	0.002
Installation of IABP or ECMO postop	79 (3.1)	6 (6.8)	0.059
Renal replacement therapy	48 (1.8)	11 (12.6)	<0.0001
Reexploration for bleeding or cardiac tamponade	63 (2.5)	12 (14.5)	<0.0001
New postoperative atrial fibrillation	373 (14.7)	21 (25.3)	0.008
Transfusion of red cell concentrates (U)	0 (0–1)	1 (0–4.5)	<0.0001
Transfusion of fresh frozen plasma (U)	0 (0–0)	0 (0–0)	<0.0001
Transfusion of platelet concentrates (U)	0 (0–0)	0 (0–0)	<0.0001
Duration of intubation (h)	5 (4–8)	6 (4–21)	0.0006
Intensive care unit stay (days)	1 (1–1.3)	1.3 (1–4)	<0.0001
Total postoperative stay (days)	9 (7–11)	11 (8–14)	0.0001
Mortality	35 (1.3)	11 (11.7)	<0.0001

Continuous variables are reported as median (first and third quartile), and categorical variables as counts and percentages, *n* (%).

CABG: coronary artery bypass grafting; ECMO: extracorporeal membrane oxygenation; IABP: intra-aortic balloon pump.

**Figure 2:**
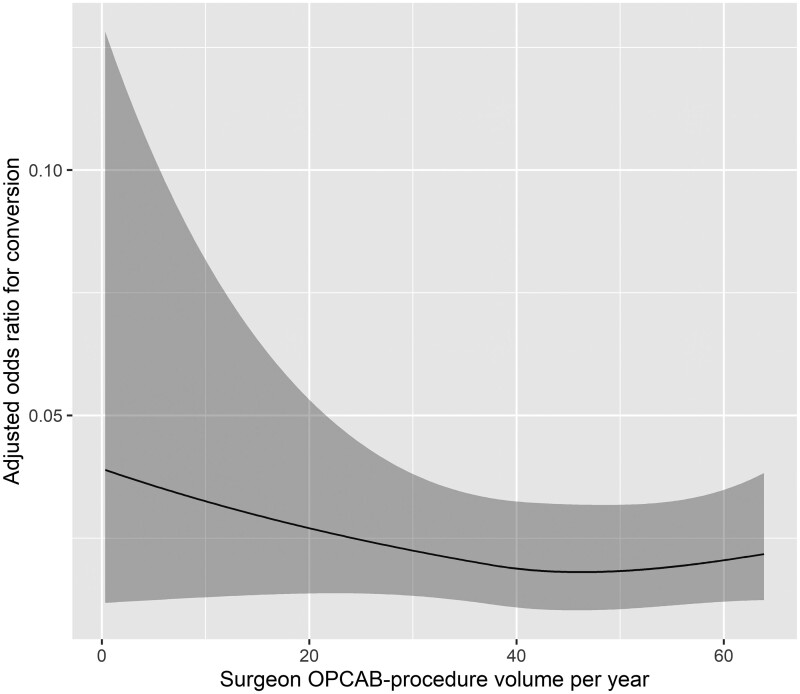
Observed relationship between surgeon OPCAB procedure volume per year and adjusted odds ratios for conversion (shaded areas present 95% confidence intervals). Curve fitting based on the results of multivariable logistic regression using restricted cubic spline transformation of the annual surgeon OPCAB procedure volume.

**Figure 3:**
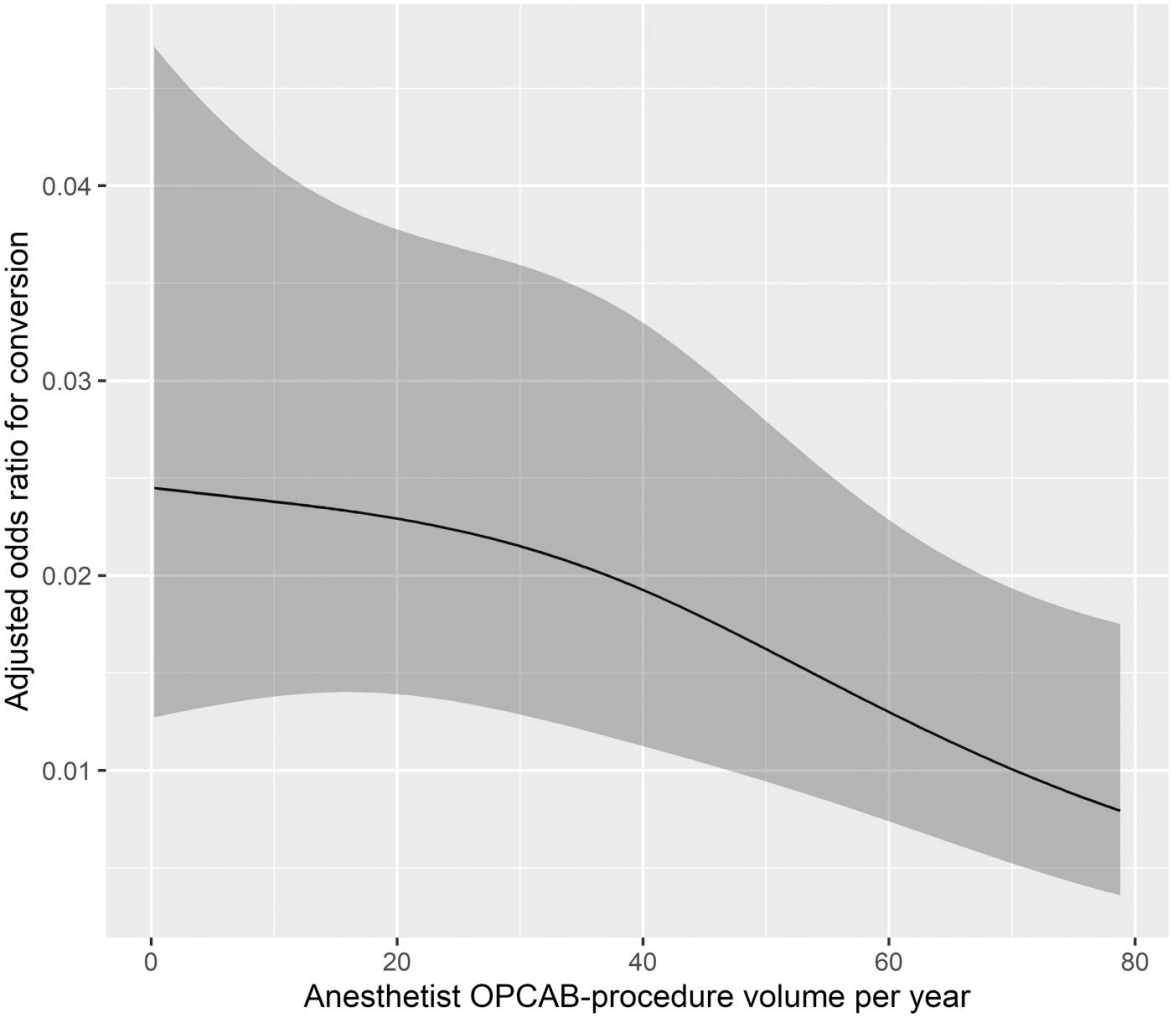
Observed relationship between anaesthetist OPCAB procedure volume per year and adjusted odds ratios for conversion (shaded areas present 95% confidence intervals). Curve fitting based on the results of multivariable logistic regression using restricted cubic spline transformation of the annual anaesthetist OPCAB procedure volume.

The results of the univariable and the multivariable logistic regression analysis for factors affecting conversion from off-pump to on-pump CABG are presented in Tables 2 and 3. Factors found to be statistically significantly associated with conversion from off-pump to on-pump CABG in the multivariable analysis were female gender [odds ratio 2.65 (95% confidence interval 1.65–4.26), *P* = 0.0001], LVEF ≤35% [odds ratio 1.91 (95% confidence interval 1.05–3.49), *P* = 0.040] and anaesthetist experience (*P* = 0.011). Lower-experience anaesthetists exhibited a higher conversion rate (Supplementary Material, Table S2). Surgeon experience was not significantly associated with conversion rate in univariable or multivariable logistic regression analysis. Figures 2 and 3 graphically present the observed relationship between surgeon/anaesthetist OPCAB procedure volume per year and adjusted odds ratios for conversion. Curve fitting was based on the results of multivariable logistic regression using restricted cubic spline transformation of the annual surgeon/anaesthetist OPCAB procedure volume. VIF analysis revealed no multicollinearity of the predictor variables of the multivariable model (range of VIF values: 1.00–1.02). CUSUM analysis has shown a conversion rate inside the calculated control limits for all anaesthetists and all but 1 surgeon who exceeded the control limit on procedure 482 out of 487 procedures (CUSUM in Supplementary Material).

**Table 2:** Univariable logistic regression analysis for factors affecting conversion from off-pump to on-pump coronary artery bypass grafting

	OR (95% CI)	*P*-Value
Age ≥65 years	1.44 (0.93–2.22)	0.095
Female gender	2.62 (1.68–4.08)	<0.0001
BMI ≥30 kg/m^2^	1.26 (0.80–1.98)	0.30
CCS class 3 or 4	1.27 (0.84–1.92)	0.25
NYHA class 3 or 4	0.79 (0.48–1.31)	0.37
Arterial hypertension	2.08 (0.95–4.55)	0.064
Dyslipidaemia	1.72 (0.86–3.46)	0.124
Diabetes mellitus	0.80 (0.50–1.28)	0.37
Current or previous smoker	0.76 (0.50–1–15)	0.198
COPD	0.66 (0.24–1.82)	0.42
Previous CVI	1.51 (0.72–3.18)	0.27
Peripheral arterial disease	0.71 (0.37–1.34)	0.29
LVEF ≤35%	2.03 (1.16–3.53)	0.012
Reduced renal function^a^	1.14 (0.65–2.01)	0.62
Previous PCI	1.54 (0.98–2.42)	0.056
Beta-blocker intake preoperatively	0.69 (0.45–1.07)	0.098
Non-elective surgery	1.25 (0.80–1.96)	0.32
Left main coronary artery stenosis (≥50%)	1.20 (0.78–1.84)	0.39
Surgeon off-pump CABG volume per year	NA^b^	0.65
Anaesthetist off-pump CABG volume per year	NA^b^	0.011

a Reduced renal function defined as blood creatinine >84 μmol/l for women and >104 μmol/l for men.

b Not available due to restricted cubic spline transformation.

BMI: body mass index; CABG: coronary artery bypass grafting; CCS: Canadian Cardiovascular Society; CI: confidence intervals; COPD: chronic obstructive pulmonary disease; CVI: cerebrovascular insult; LVEF: left ventricular ejection fraction; NA: not available; NYHA: New York Heart Association; OR: odds ratio; PCI: percutaneous coronary intervention.

**Table 3:** Multivariable logistic regression analysis for factors affecting conversion from off-pump to on-pump coronary artery bypass grafting

	OR (95% CI)	*P*-value
Age ≥65 years	1.27 (0.80–2.03)	0.29
Female gender	2.65 (1.65–4.26)	0.0001
LVEF ≤35%	1.91 (1.05–3.49)	0.040
Non-elective surgery	1.20 (0.74–1.94)	0.48
Left main coronary artery stenosis (≥50%)	1.23 (0.79–1.92)	0.42
Surgeon off-pump CABG volume per year	NA^a^	0.51
Anaesthetist off-pump CABG volume per year	NA^a^	0.011

a Not available due to restricted cubic spline transformation.

CABG: coronary artery bypass grafting; CI: confidence intervals; LVEF: left ventricular ejection fraction; NA: not available; OR: odds ratio.

## DISCUSSION

Analyzing the data of a large single-centre cohort of patients undergoing CABG, initially started as off-pump, we found a conversion to on-pump rate of 3.4% (only 0.02% with cardioplegic arrest). Non-elective conversion to on-pump CABG was associated with worse in-hospital postoperative outcomes and converted patients had a higher rate of postoperative cardiopulmonary resuscitation, renal replacement therapy, re-exploration for bleeding or cardiac tamponade, blood product transfusion, longer intubation duration, longer intensive care unit stay, longer postoperative hospital stay and higher mortality in comparison to non-converted patients.

Patient caseload is an important but not the sole factor for conversion from off-pump to on-pump CABG. To address this issue, we have performed a univariable logistic regression analysis to assess the impact of several other factors on the rate of conversion from off-pump to on-pump CABG. Advanced age, low LVEF, preoperative intra-aortic balloon pump requirement, increasing number of diseased coronary arteries, urgent surgery, preoperative use of beta-blockers, chronic atrial fibrillation, previous CABG and preoperative acute myocardial infarction are some of the factors that published studies have found to be associated with conversion from off-pump to on-pump CABG. Female gender is another risk factor for conversion, probably associated with advanced age at clinical presentation and small coronary arteries [9–14, 16]. In our study, we found female gender, LVEF ≤35% and low anaesthetist experience to be associated with a high risk for conversion from off-pump to on-pump CABG. Finally, we have performed a comprehensive multivariable logistic regression analysis to adjust surgeon- and anaesthetist experience for several risk factors for conversion. Anaesthetist experience remained a significant risk factor for conversion after multivariable adjustment.

Intraoperative conversion from off-pump to on-pump CABG may occur in 1.4–16% of off-pump CABG procedures, and our conversion rate lies near the lower end of the conversion rate range observed in the published literature [8–13]. In a large nationwide evaluation of the Society of Thoracic Surgeons database including 196 576 patients undergoing planned off-pump CABG, an overall conversion rate of 5.5% was observed. Patients undergoing conversion had higher morbidity and mortality than off-pump patients, with non-elective conversions for haemodynamic instability having even worse outcomes, exhibiting nearly 4 times greater in-hospital mortality than patients who underwent off-pump CABG [9]. Analysis of a large single-centre database, including 5353 patients undergoing planned off-pump CABG in a high-volume off-pump CABG centre by a team of dedicated off-pump CABG surgeons, showed an overall conversion rate of 1.4%. This is the lowest overall conversion rate of all published studies and potentially indicates the positive impact of high surgeon experience in the early detection of intraoperative haemodynamic instability and subsequent avoidance of non-elective intraoperative conversions. Likewise, in the same study, no difference in postoperative mortality was observed between the converted and the non-converted patients in propensity score matched groups, even though the crude mortality rates were higher in the converted group [8].

Surgeon experience is of critical importance for the achievement of optimal results in off-pump CABG, as underlined in a consensus conference and statement paper of the International Society for Minimally Invasive Cardiothoracic Surgery and is associated with conversion from off-pump to on-pump CABG [7, 10, 16]. In our study, all procedures were performed by a total of 12 surgeons. Our data did not show any effect of surgeon experience on conversion. CUSUM analysis for surgeons has shown an overall constant conversion rate over time, with only 1 surgeon exceeding the CUSUM control limit during the latest procedures of the study period. It should be noted, however, that in our centre, isolated CABG procedures are almost exclusively performed in off-pump technique, so the overall experience with the off-pump CABG procedure is high.

Off-pump CABG is a team effort where timely communication and interplay between surgeon and anaesthetist is of paramount importance. Despite the significance of anaesthetist experience on the feasibility of off-pump CABG and the potential avoidance of non-elective conversion, no published studies have examined the impact of this factor on the conversion rate. In our study, off-pump CABG was supported by a total of 24 anaesthetists. Our data have shown an effect of anaesthetist experience on conversion, with less experienced anaesthetists exhibiting higher conversion rates. These results underline the importance of anaesthetist experience in the avoidance of conversion. Anaesthetists are the team members primarily assessing haemodynamics while the surgeon is performing the coronary anastomoses, so that their judgement is critical to prevent conversions. CUSUM analysis for anaesthetists has shown an overall constant conversion rate over time with no anaesthetist exceeding the CUSUM control limit during the entire study period.

Based on our data, non-elective conversion is associated with a significant increase in perioperative morbidity and mortality, which may be caused either directly by the haemodynamic instability and tissue hypoperfusion surrounding non-elective conversion or may reflect patient-specific factors. Primary on-pump procedure in these patients might have been associated with lower risk; however, it is hard to predict which patients will require non-elective conversion, in order to avoid primary off-pump CABG. Even though conversion from off-pump to on-pump surgery does not seem to be completely preventable, careful intraoperative monitoring of end-tidal carbon dioxide pressure using capnography may be helpful to assess cardiac output trends after repositioning the heart, especially when grafting the posterior and lateral coronary vessels in order to prevent this complication. Continually falling end-tidal carbon dioxide pressure has been found to be associated with decreased cardiac output and may precede haemodynamic instability leading to conversion [8, 17].

Further analysis of our data, following the division of the 15-year study period into 3 equal subperiods, showed successively increasing conversion rate from the first to third subperiod without increasing mortality. The observed increasing conversion rate was not associated with increasing number of high-risk patients, as reflected by significantly lower additive EuroSCORE in the second and third study subperiods, therefore, should be most probably attributed to decreasing threshold for conversion in the later periods.

### Limitations

This study has limitations associated with the retrospective data analysis and its inherent selection bias, as well as confounding variables which might not have been accounted for during this analysis (e.g. use of intra-aortic balloon pump). Even though individual surgeon-related factors and seamless manual technical skills are of utmost importance for off-pump CABG, no objective assessment of these skills was available in our study. The decision to convert from off-pump to on-pump CABG was according to the judgement of both surgeon and anaesthetist and was performed on a one-by-one basis. Unfortunately, no information is available regarding the variability of the processes, whereon the decision to convert was based. However, the main strengths of this study must also be emphasized. This is a large single-centre cohort including 2742 patients operated on during a 15-year time-span, which is a considerable off-pump CABG experience. Additionally, to our knowledge, this is the first published study assessing the impact of anaesthetist experience on the conversion rate from off-pump to on-pump CABG. Finally, our conclusions arise from a comprehensive multivariable logistic regression analysis, where surgeon- and anaesthetist experience was adjusted for several well-known risk factors for conversion.

## CONCLUSION

To conclude, non-elective intraoperative conversion from off-pump to on-pump CABG is associated with worse in-hospital postoperative outcomes reflected in higher morbidity and mortality. Increased alertness for haemodynamic instability preceding conversion is essential to avoid the detrimental effects of non-elective conversion to on-pump CABG. Considering the strengths and limitations outlined previously, our data suggest that limited experience of anaesthetists in off-pump CABG is associated with a higher conversion rate.

## Supplementary Material

ivad205_Supplementary_Data

## Data Availability

The data that support the findings of this study are available from the corresponding author, upon reasonable request.

## Notes

Presented at the Annual Meeting of the European Association for Cardio-Thoracic Surgery, Lisbon, Portugal, 4 October 2019.

## ABBREVIATIONS

CABG: Coronary artery bypass grafting
CUSUM: Cumulative sum control chart
EuroSCORE: European system for cardiac operative risk evaluation
LVEF: Left ventricular ejection fraction

## References

[1] Myers WO , Blackstone EH, Davis K, Foster ED, Kaiser GC. CASS Registry long term surgical survival. Coronary Artery Surgery Study. J Am Coll Cardiol 1999;33:488–98. 9973030 10.1016/s0735-1097(98)00563-4

[2] Head SJ , Milojevic M, Daemen J, Ahn JM, Boersma E, Christiansen EH et al Mortality after coronary artery bypass grafting versus percutaneous coronary intervention with stenting for coronary artery disease: a pooled analysis of individual patient data. Lancet (London, England) 2018;391:939–48. 29478841 10.1016/S0140-6736(18)30423-9

[3] Gaudino M , Hameed I, Farkouh ME, Rahouma M, Naik A, Robinson NB et al Overall and cause-specific mortality in randomized clinical trials comparing percutaneous interventions with coronary bypass surgery: a meta-analysis. JAMA Intern Med 2020;180:1638–46. 33044497 10.1001/jamainternmed.2020.4748 PMC7551235

[4] Deppe AC , Arbash W, Kuhn EW, Slottosch I, Scherner M, Liakopoulos OJ et al Current evidence of coronary artery bypass grafting off-pump versus on-pump: a systematic review with meta-analysis of over 16,900 patients investigated in randomized controlled trials. Eur J Cardiothorac Surg 2016;49:1031–41; discussion 1041. 26276839 10.1093/ejcts/ezv268

[5] Altarabsheh SE , Deo SV, Rababa'h AM, Lim JY, Cho YH, Sharma V et al Off-pump coronary artery bypass reduces early stroke in octogenarians: a meta-analysis of 18,000 patients. Ann Thorac Surg 2015;99:1568–75. 25791924 10.1016/j.athoracsur.2014.12.057

[6] Khan H , Uzzaman M, Benedetto U, Butt S, Raja SG. On- or off-pump coronary artery bypass grafting for octogenarians: a meta-analysis of comparative studies involving 27,623 patients. Int J Surg 2017;47:42–51. 28951288 10.1016/j.ijsu.2017.09.054

[7] Puskas JD , Martin J, Cheng DC, Benussi S, Bonatti JO, Diegeler A et al ISMICS consensus conference and statements of randomized controlled trials of off-pump versus conventional coronary artery bypass surgery. Innovations 2015;10:219–29. 26371452 10.1097/IMI.0000000000000184

[8] Pullan M , Oo A, Poullis M. Off-pump conversion: in-hospital mortality and long-term survival. Thorac Cardiovasc Surg 2017;65:296–301. 26600406 10.1055/s-0035-1566742

[9] Keeling B , Thourani V, Aliawadi G, Kim S, Cyr D, Badhwar V et al Conversion from off-pump coronary artery bypass grafting to on-pump coronary artery bypass grafting. Ann Thorac Surg 2017;104:1267–74. 28610886 10.1016/j.athoracsur.2017.03.032

[10] Stevens LM , Noiseux N, Avezum A, Ayapati DR, Chen X, Lucchese FA et al; CORONARY Investigators. Conversion after off-pump coronary artery bypass grafting: the CORONARY trial experience. Eur J Cardiothorac Surg 2017;51:539–46. 28082464 10.1093/ejcts/ezw361

[11] Yoon SS , Bang JH, Jeong SS, Jeong JH, Woo JS. Risk factors of on-pump conversion during off-pump coronary artery bypass graft. Korean J Thorac Cardiovasc Surg 2017;50:355–62. 29124027 10.5090/kjtcs.2017.50.5.355 PMC5628963

[12] Lim J , Lee WY, Ra YJ, Jeong JH, Ko HH. Analysis of risk factors for conversion from off-pump to on-pump coronary artery bypass graft. Korean J Thorac Cardiovasc Surg 2017;50:14–21. 28180098 10.5090/kjtcs.2017.50.1.14 PMC5295478

[13] Chakravarthy M , Prabhakumar D, Patil TA, George A, Jawali V. Conversion during off-pump coronary artery bypass graft surgery: a case-control study. Ann Card Anaesth 2019;22:18–23. 30648674 10.4103/aca.ACA_227_17 PMC6350426

[14] Chowdhury R , White D, Kilgo P, Puskas JD, Thourani VH, Chen EP et al Risk factors for conversion to cardiopulmonary bypass during off-pump coronary artery bypass surgery. Ann Thorac Surg 2012;93:1936–41; discussion 1942. 22503849 10.1016/j.athoracsur.2012.02.051

[15] Ueki C , Yamamoto H, Motomura N, Miyata H, Sakata R, Tsuneyoshi H. Effect of hospital and surgeon procedure volumes on the incidence of intraoperative conversion during off-pump coronary artery bypass grafting. Semin Thorac Cardiovasc Surg 2021;33:49–58. 33242613 10.1053/j.semtcvs.2020.08.019

[16] Edgerton JR , Dewey TM, Magee MJ, Herbert MA, Prince SL, Jones KK et al Conversion in off-pump coronary artery bypass grafting: an analysis of predictors and outcomes. Ann Thorac Surg 2003;76:1138. 14530000 10.1016/s0003-4975(03)00747-1

[17] Saleh HZ , Pullan DM. Monitoring cardiac output trends with end-tidal carbon dioxide pressures in off-pump coronary bypass. Ann Thorac Surg 2011 ;91:e81–2. 21524440 10.1016/j.athoracsur.2010.12.039

