# Supplementary material for: Conversion from off-pump to on-pump coronary artery bypass grafting: impact of surgeon and anaesthetist experience
Source: Interdiscip Cardiovasc Thorac Surg. 2023 Dec 20;37(6):ivad205. doi: 10.1093/icvts/ivad205 (PMC10751234; doi:10.1093/icvts/ivad205)
Supplement: ivad205_Supplementary_Data [file ivad205_supplementary_data.docx]

**Supplemental Material:**

1. **CUSUM charts of individual surgeons**
2. **CUSUM charts of individual anesthetists**
3. **Supplemental table 1. Number of conversions and procedures for individual surgeons.**
4. **Supplemental table 2. Number of conversions and procedures for individual anesthetists.**
5. **CUSUM charts of individual surgeons**
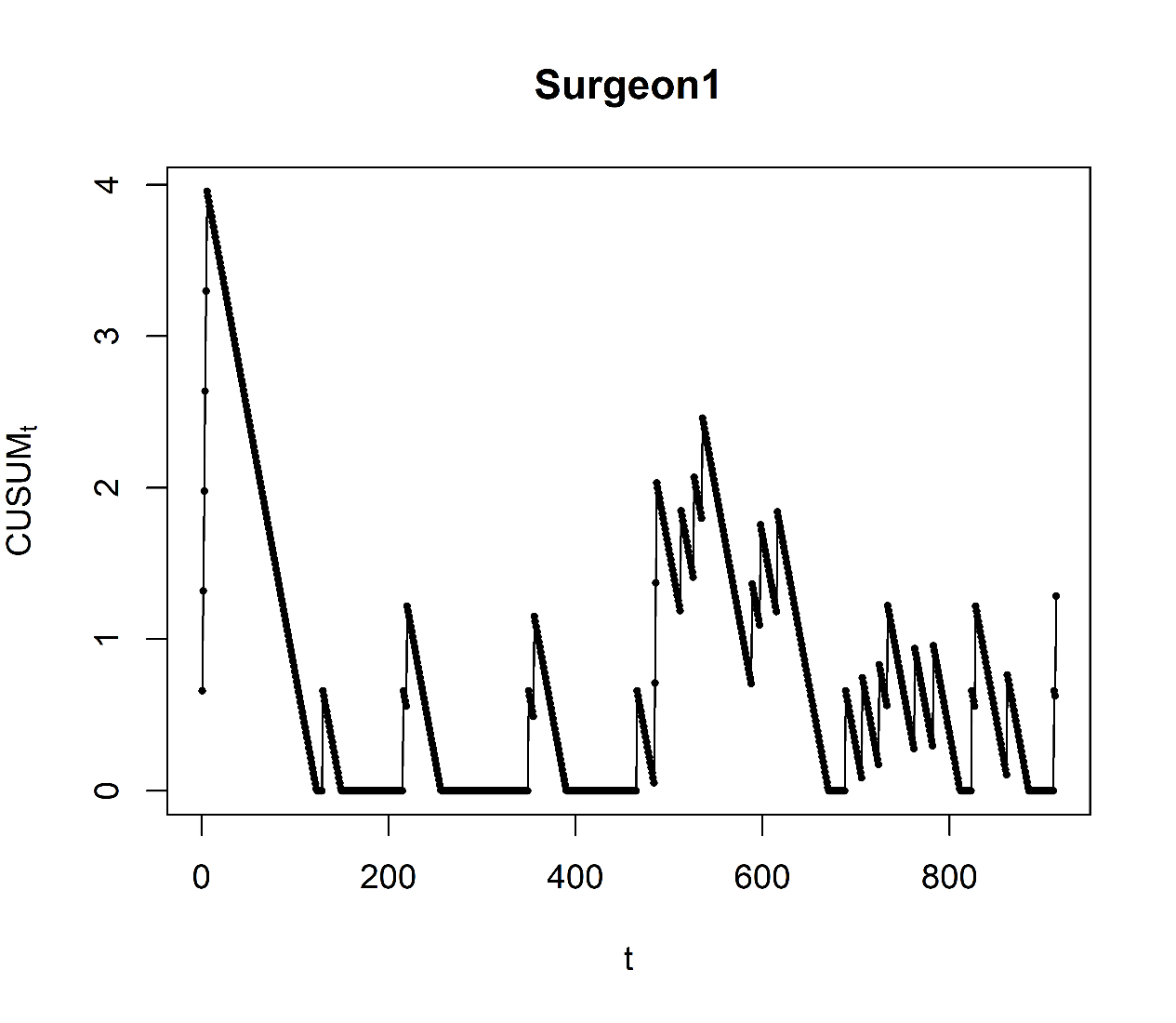

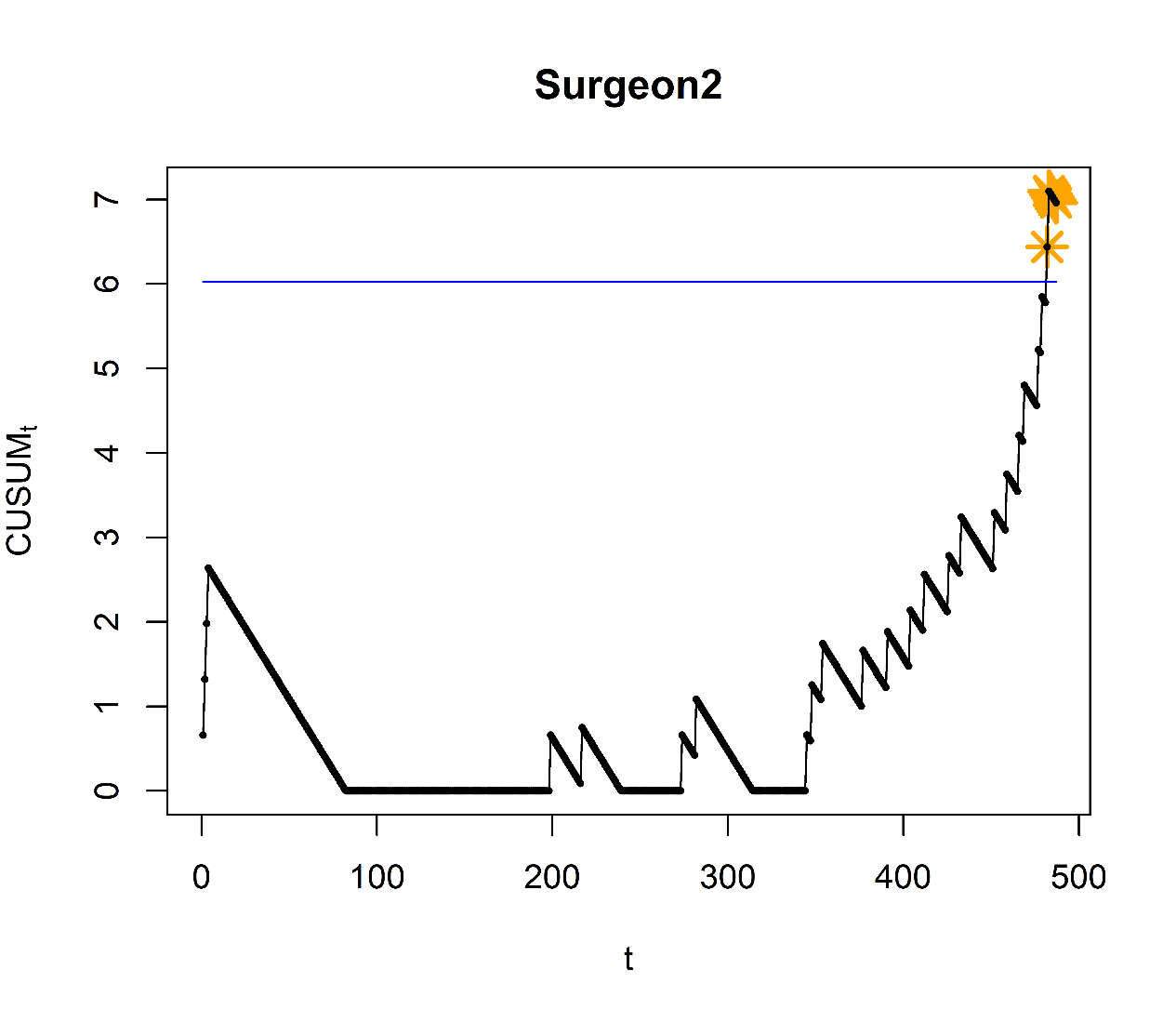

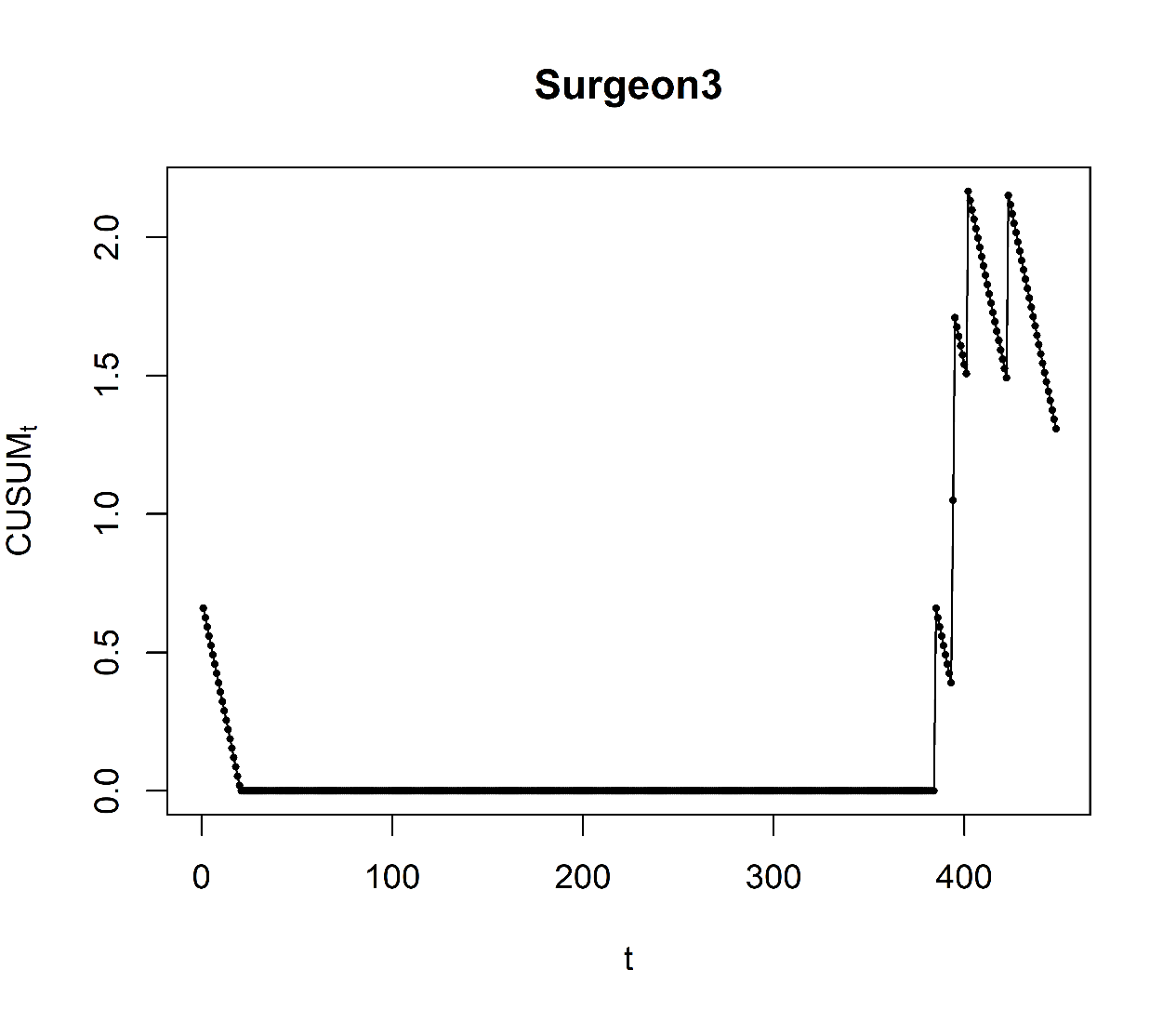

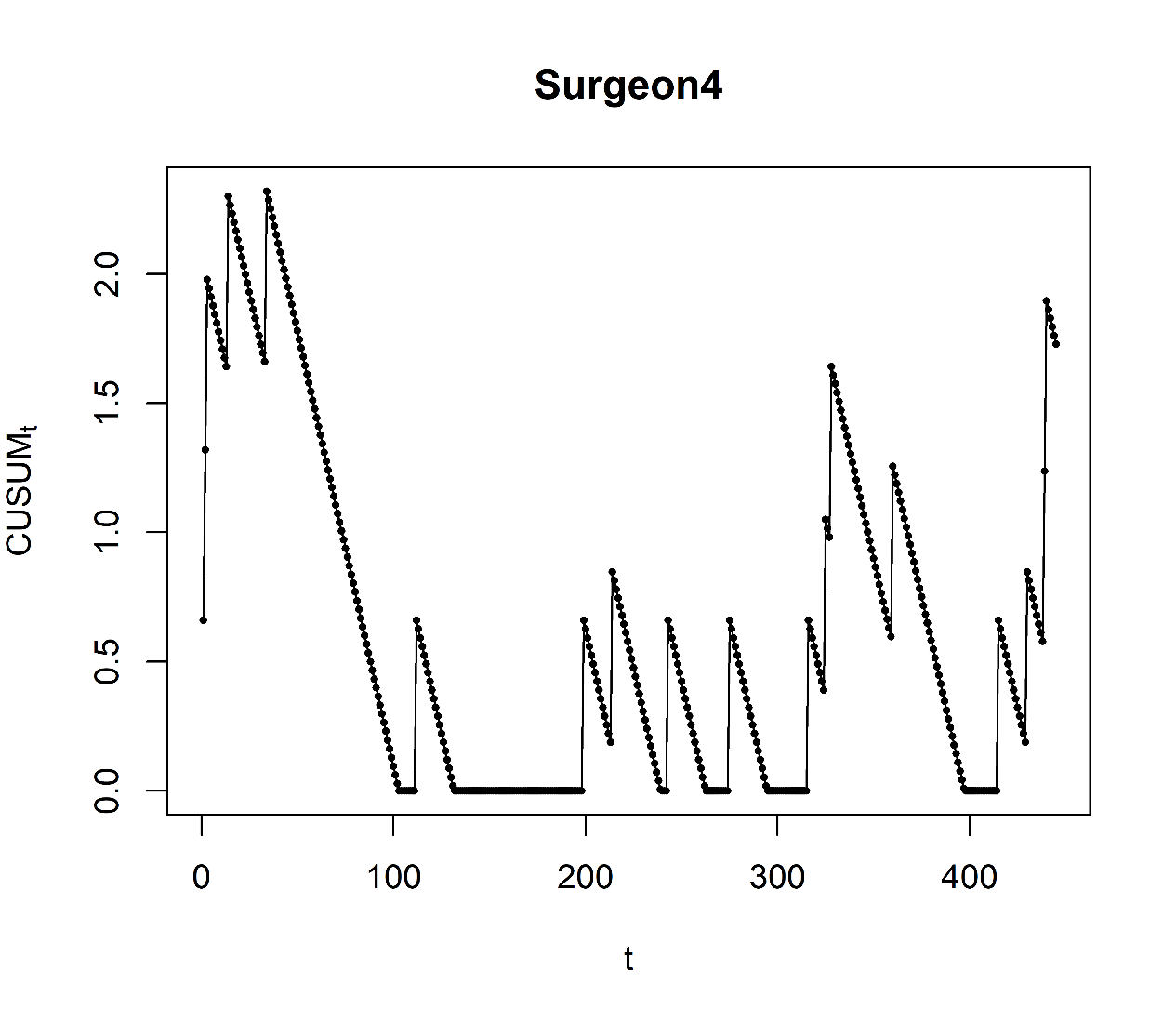

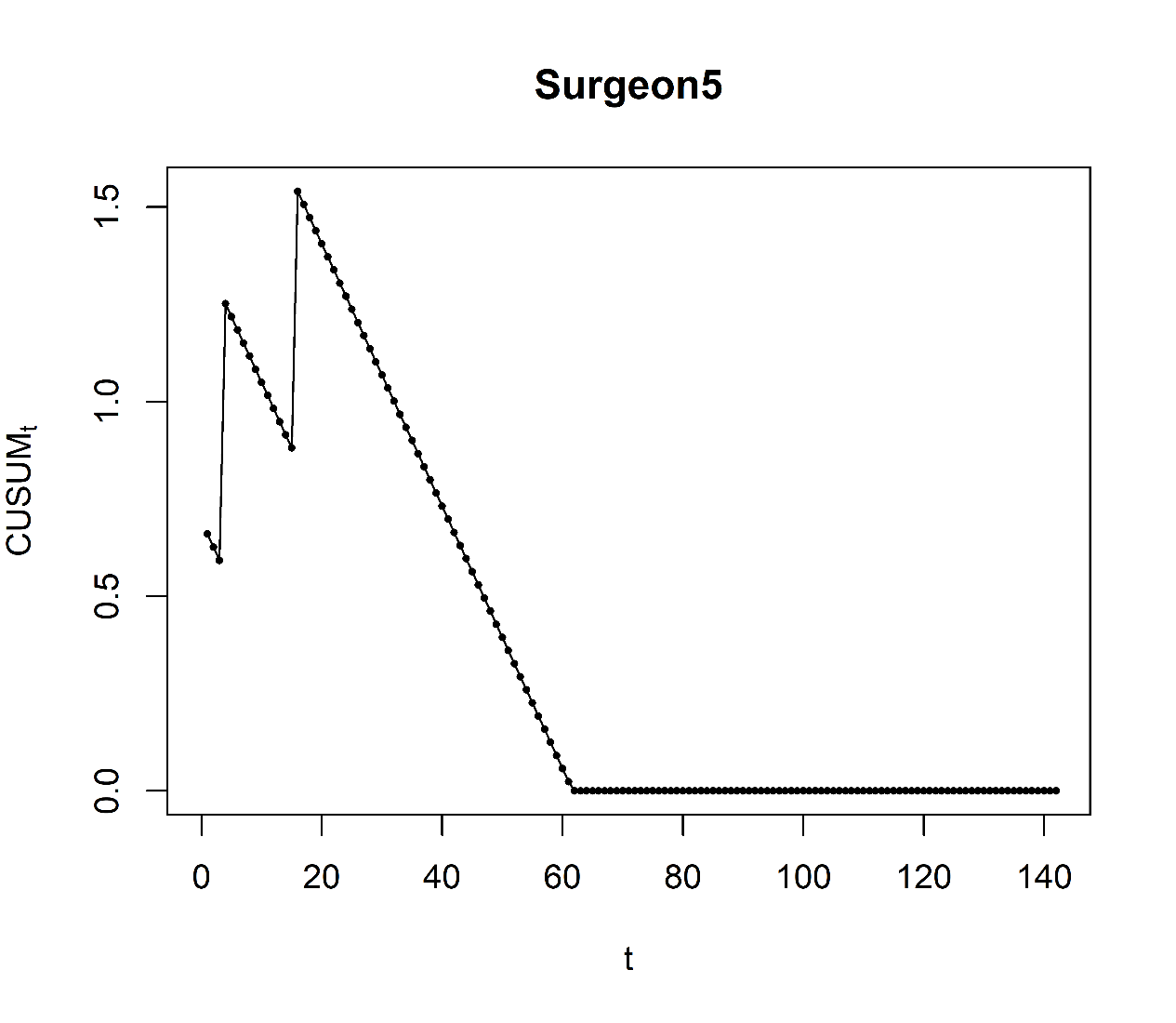

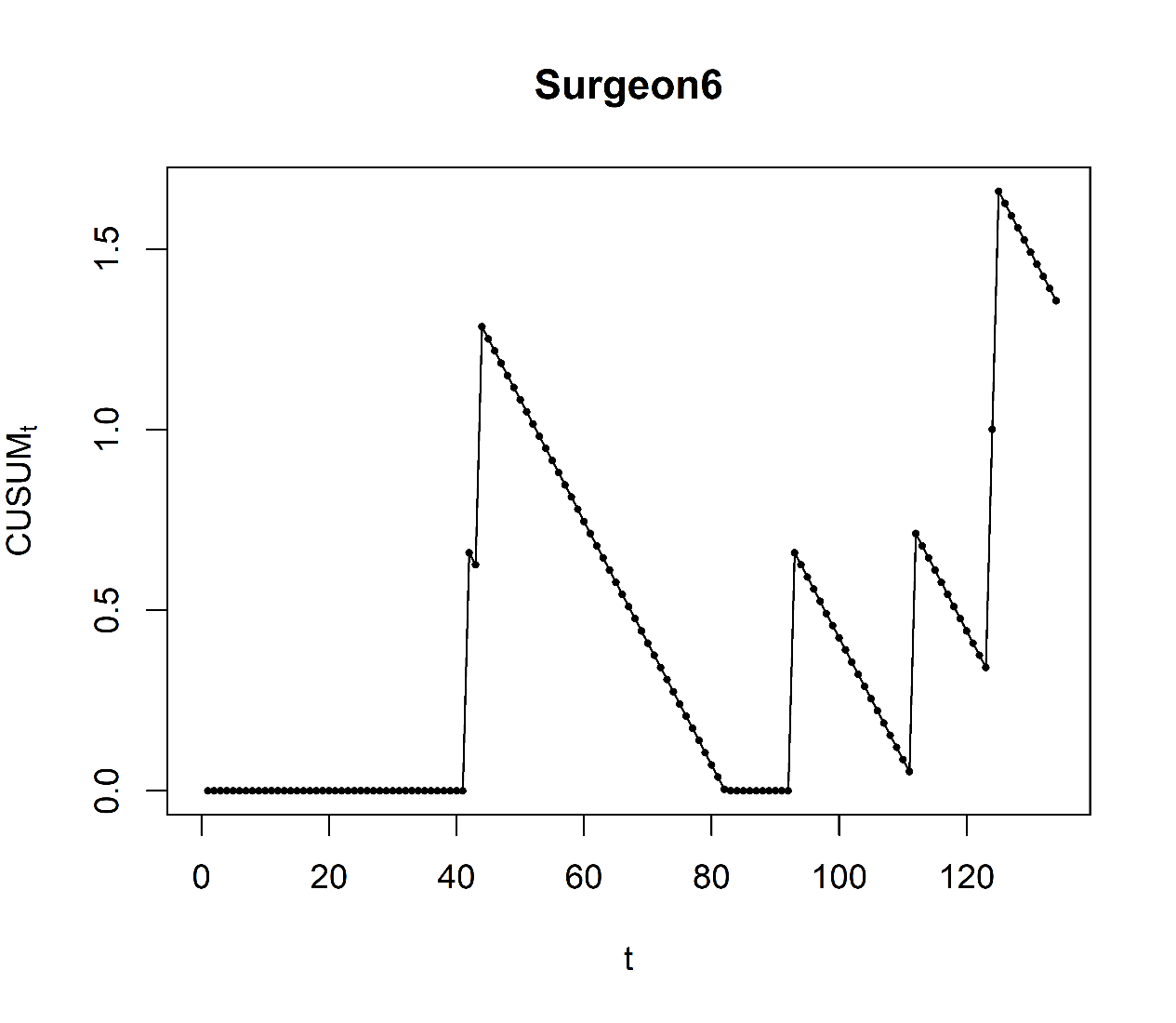

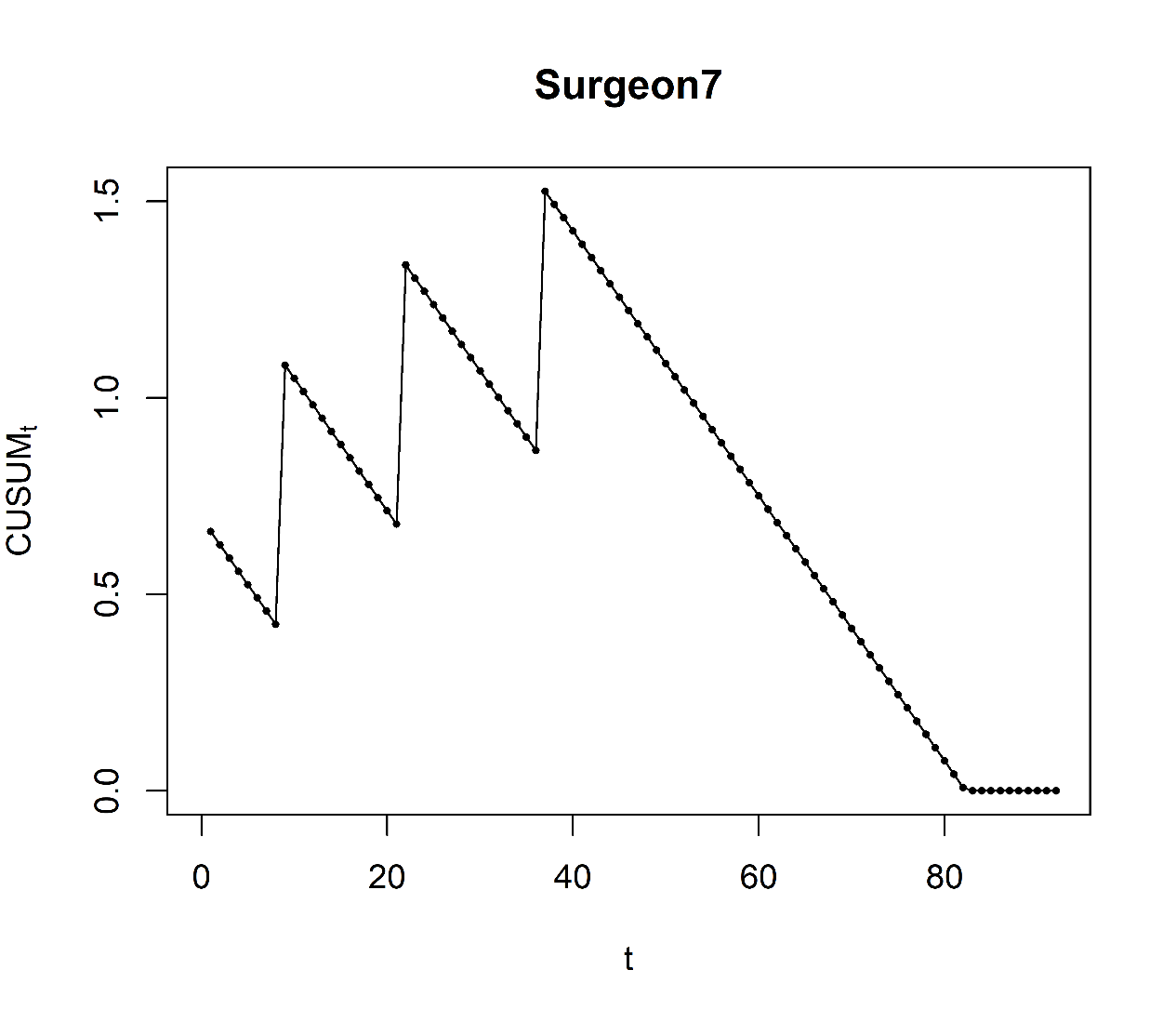

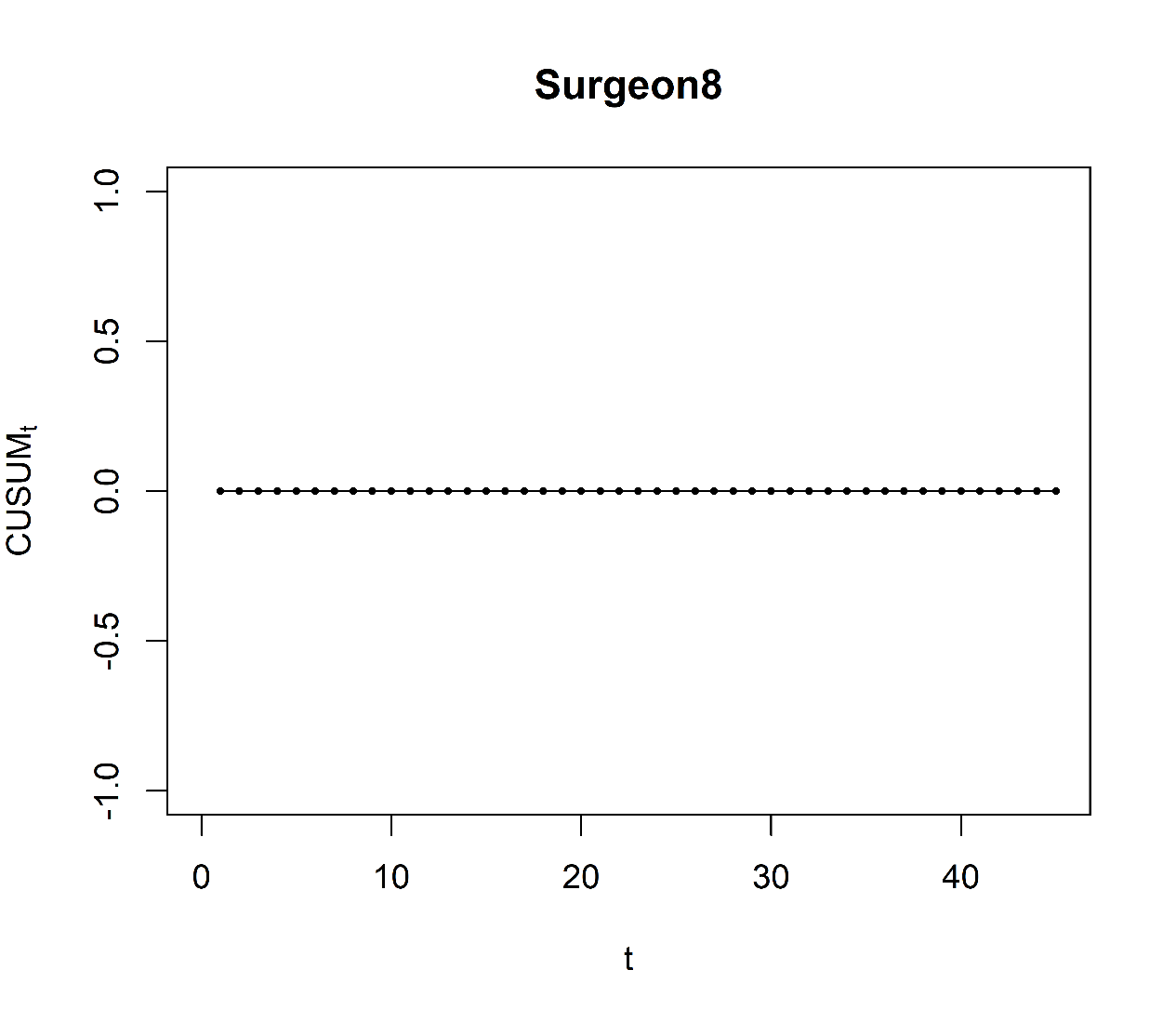

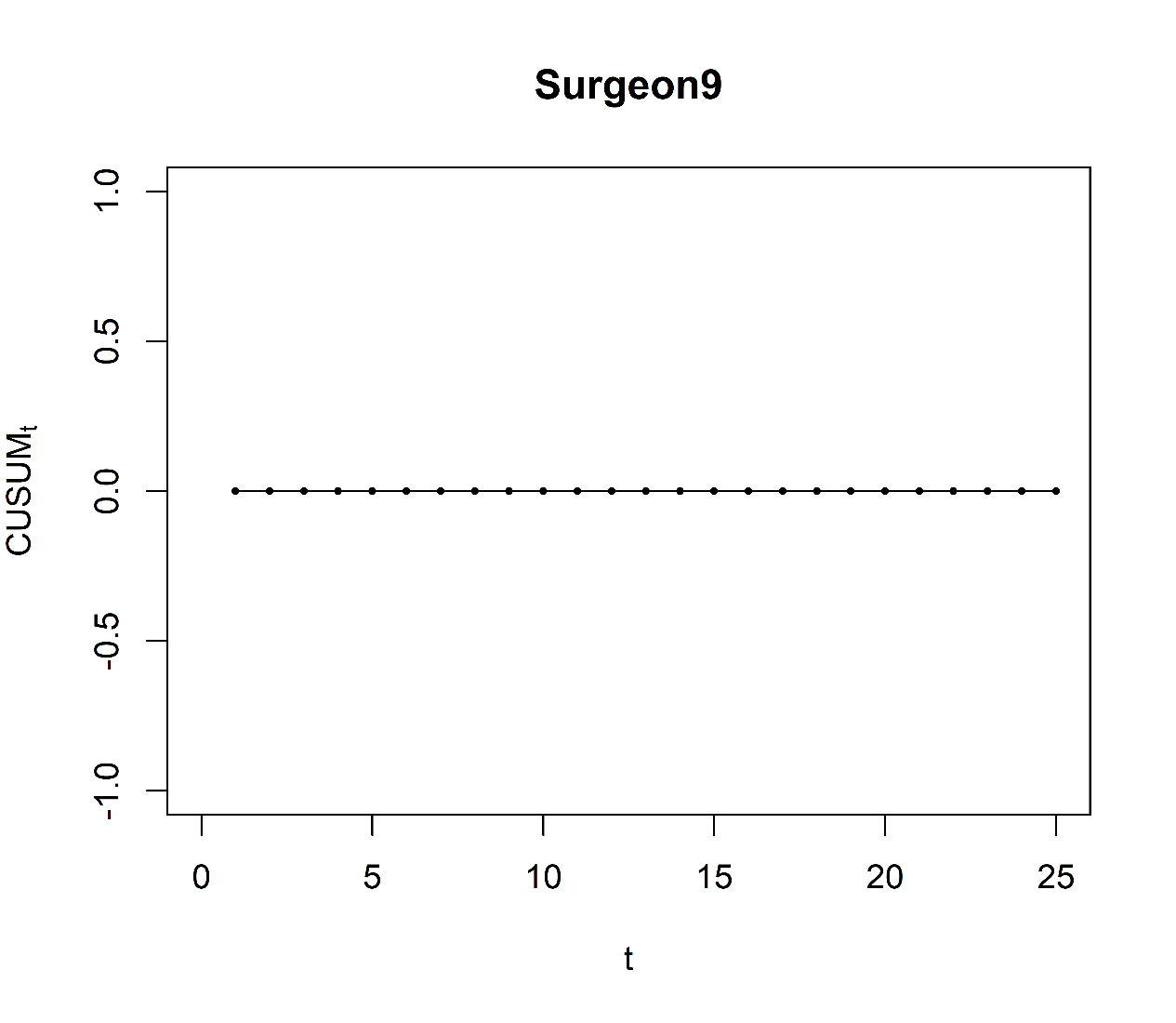

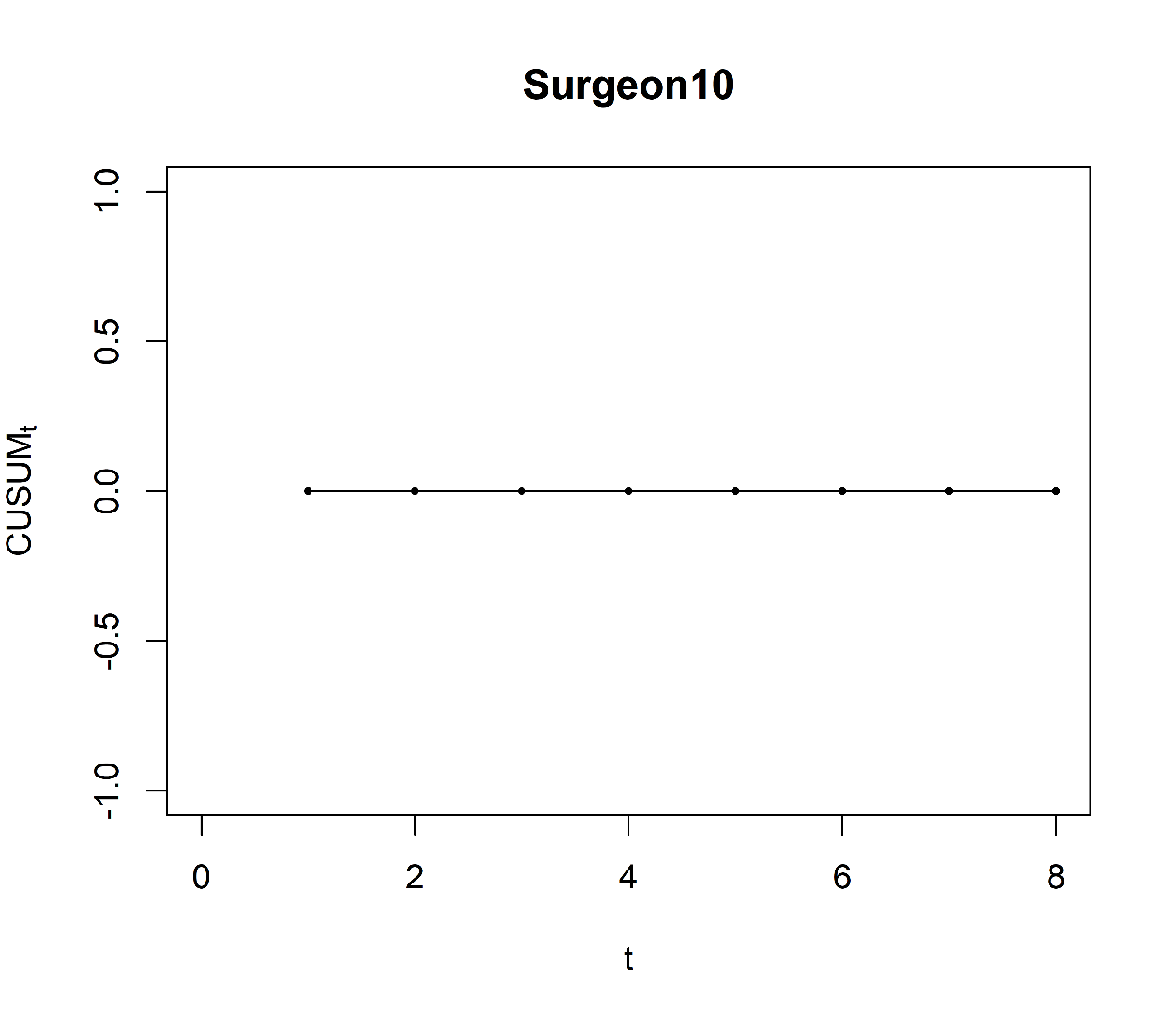

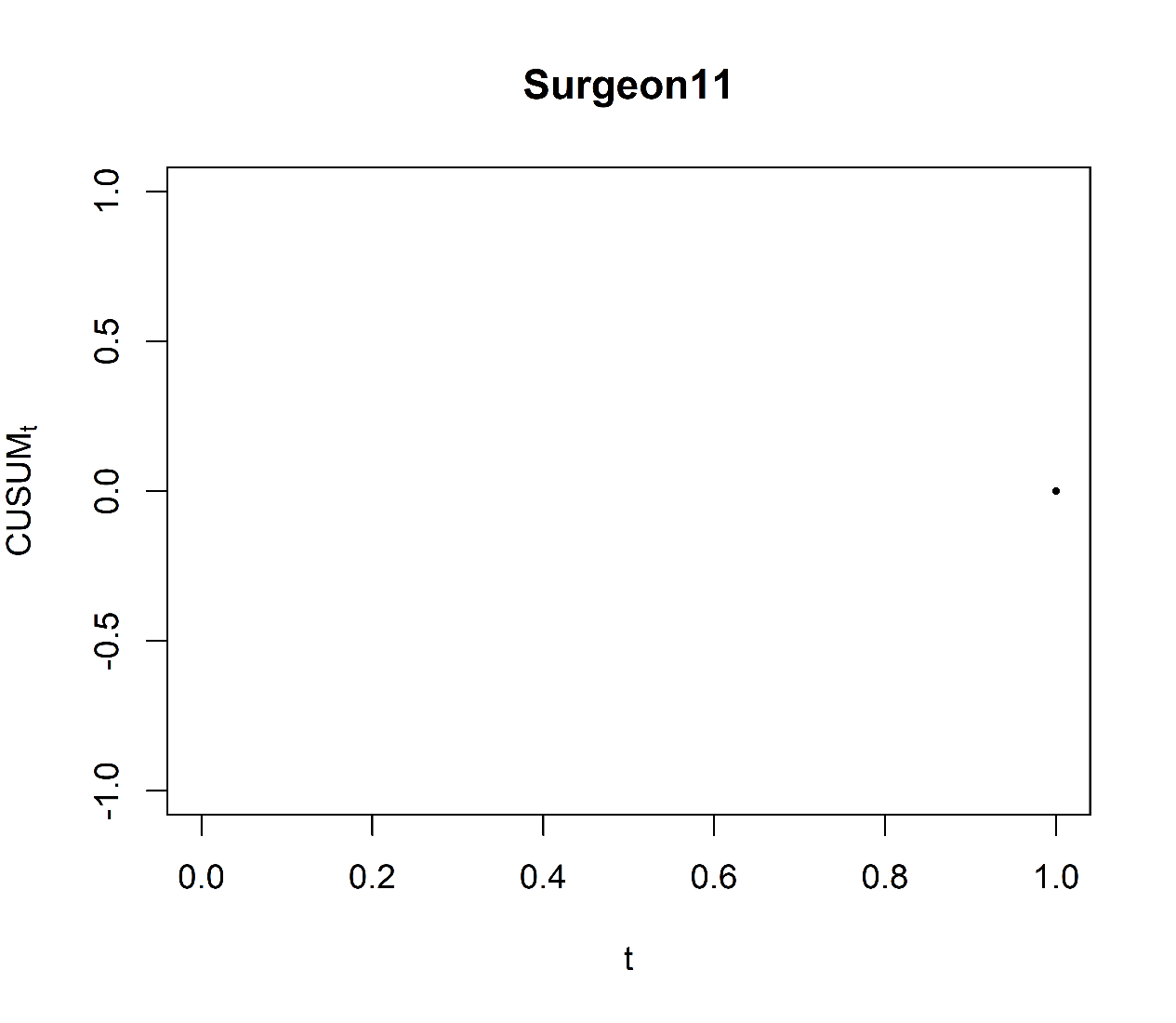

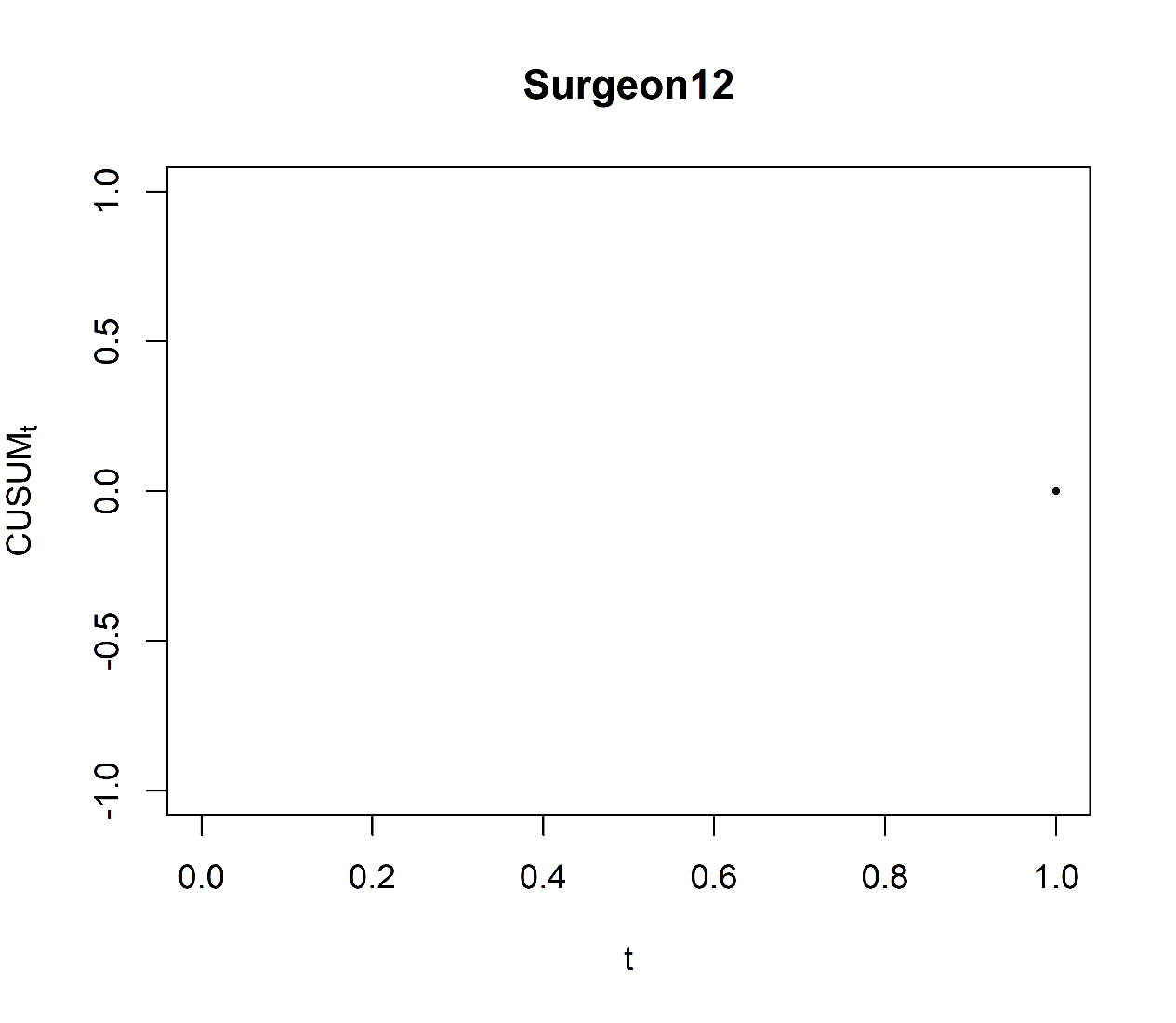

6. **CUSUM charts of individual anesthetists**
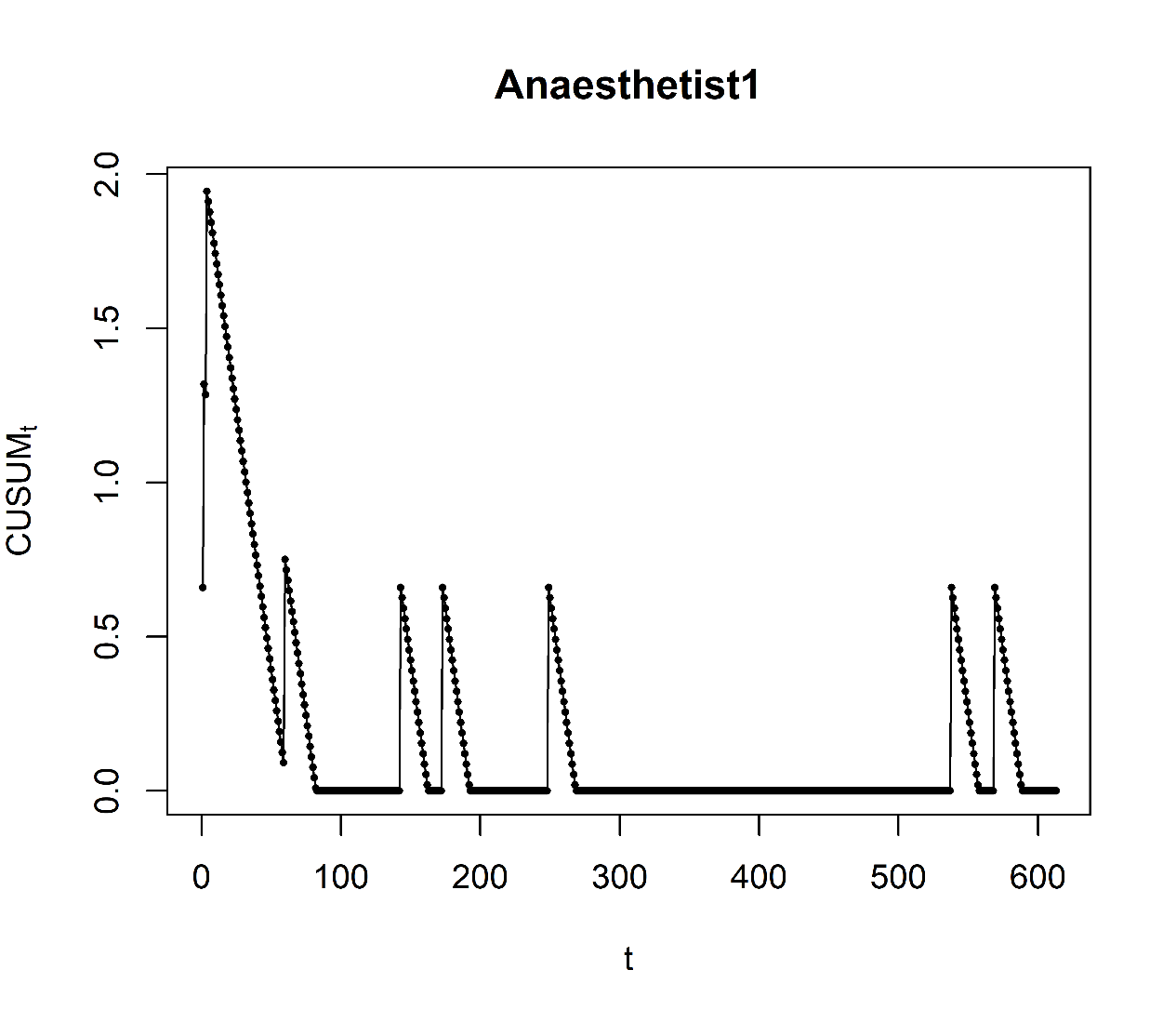

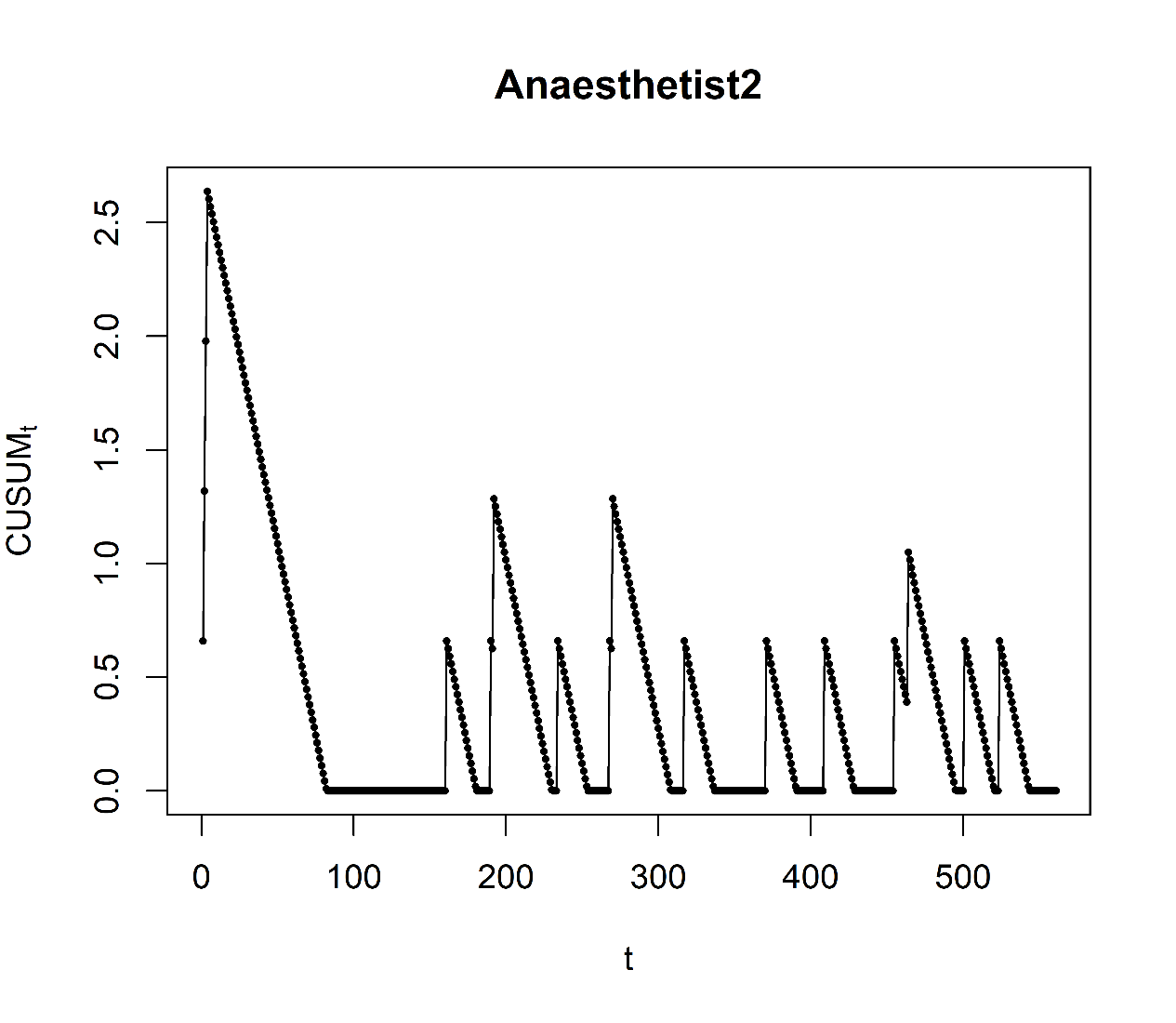

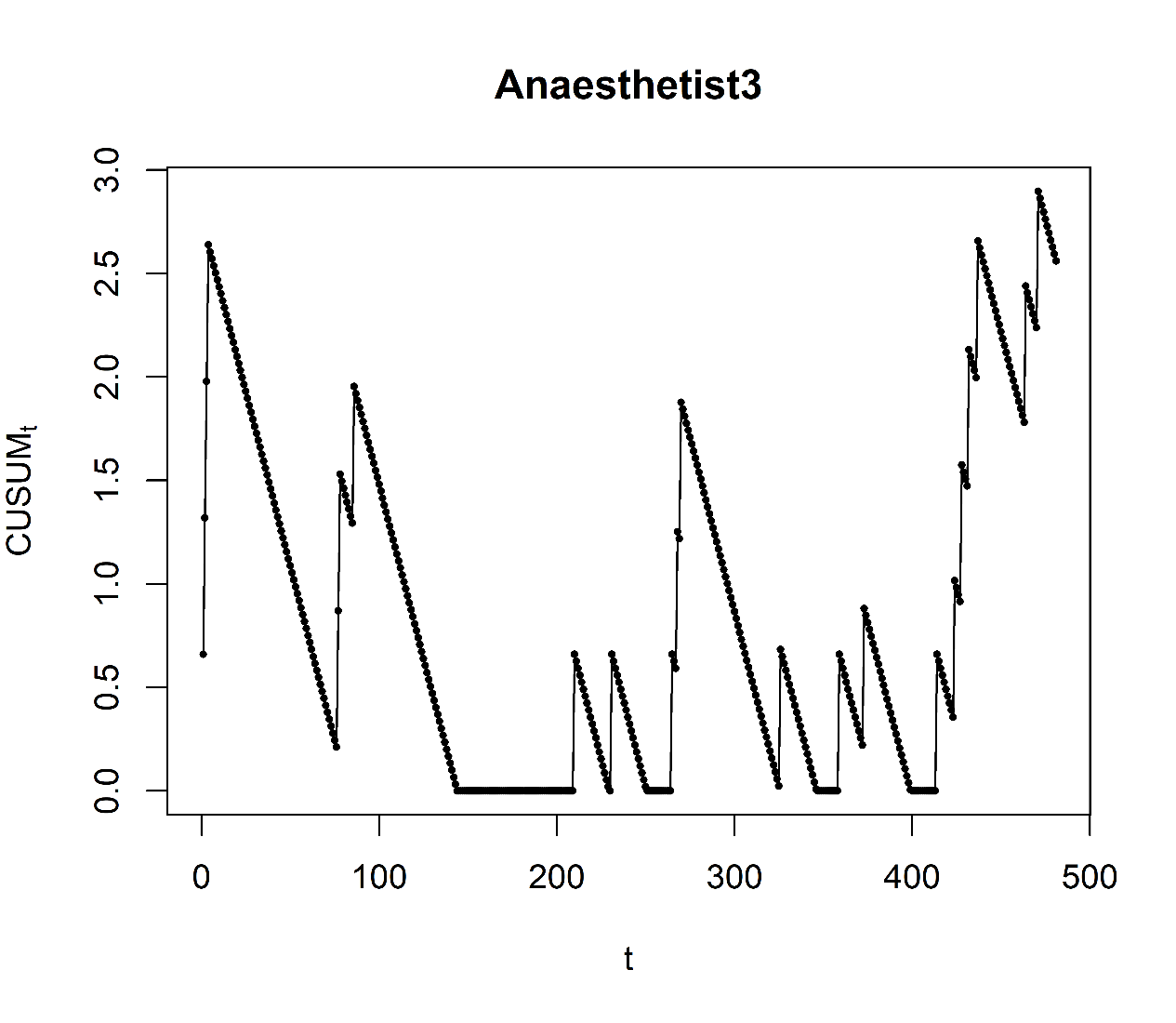

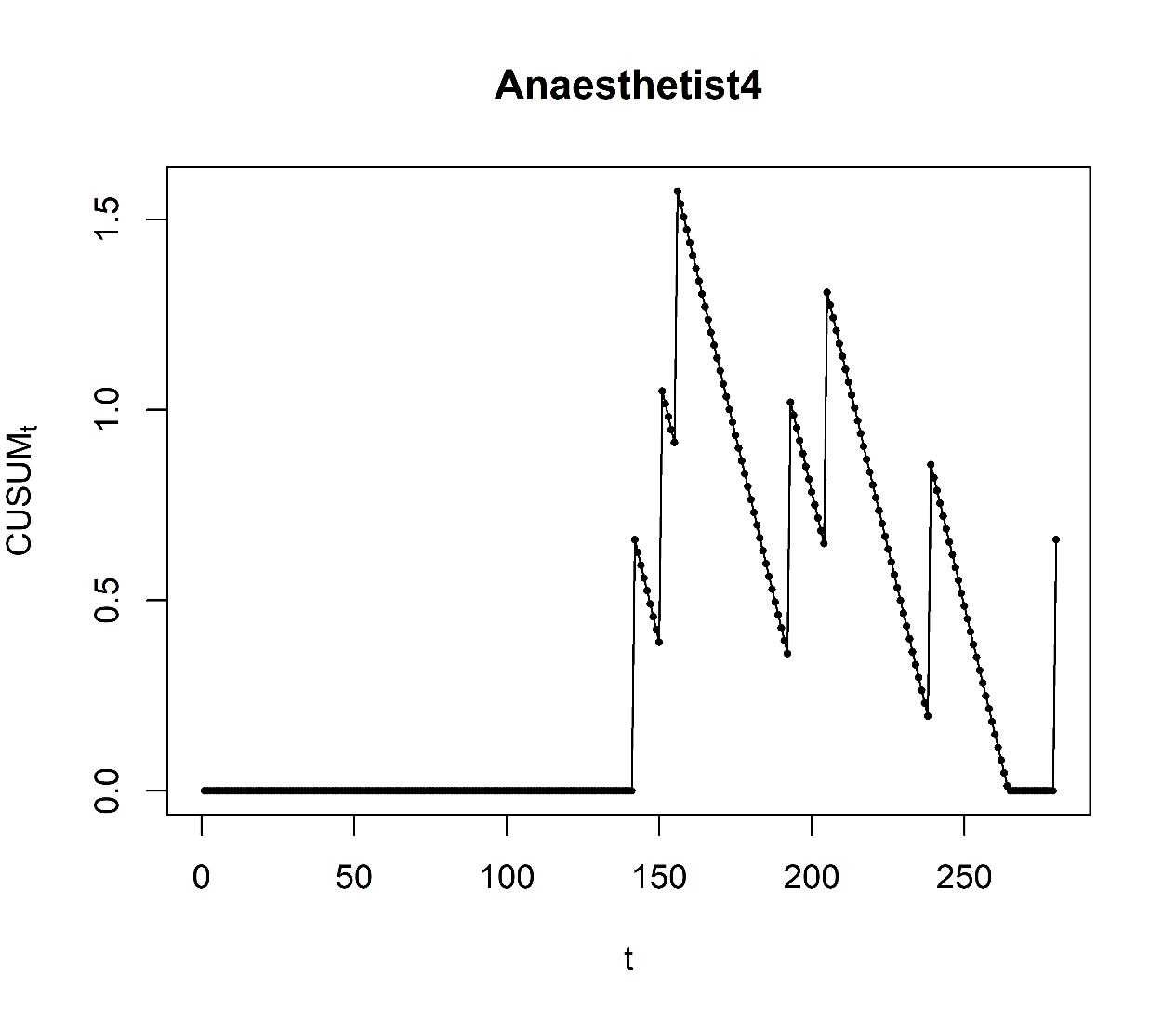

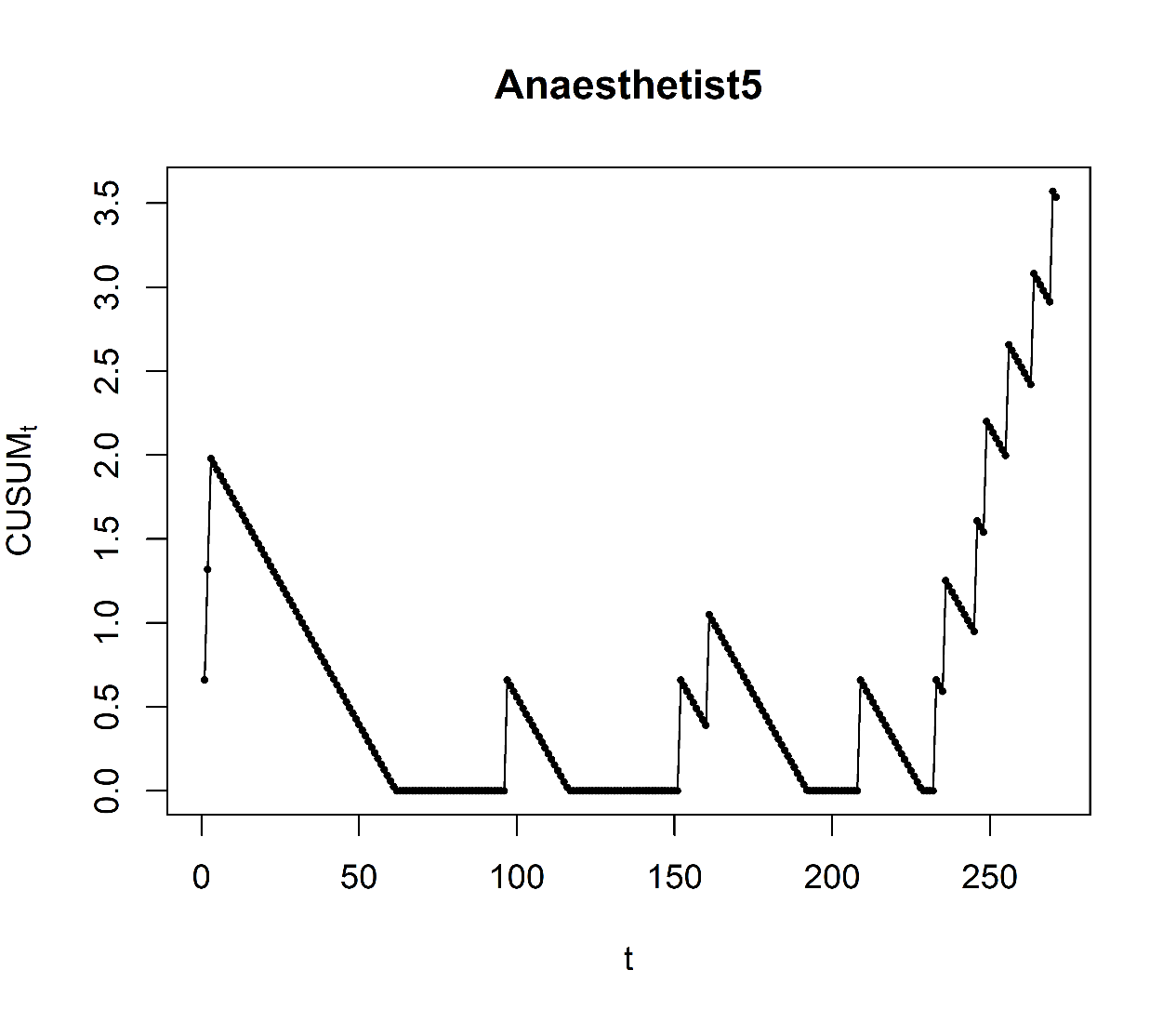

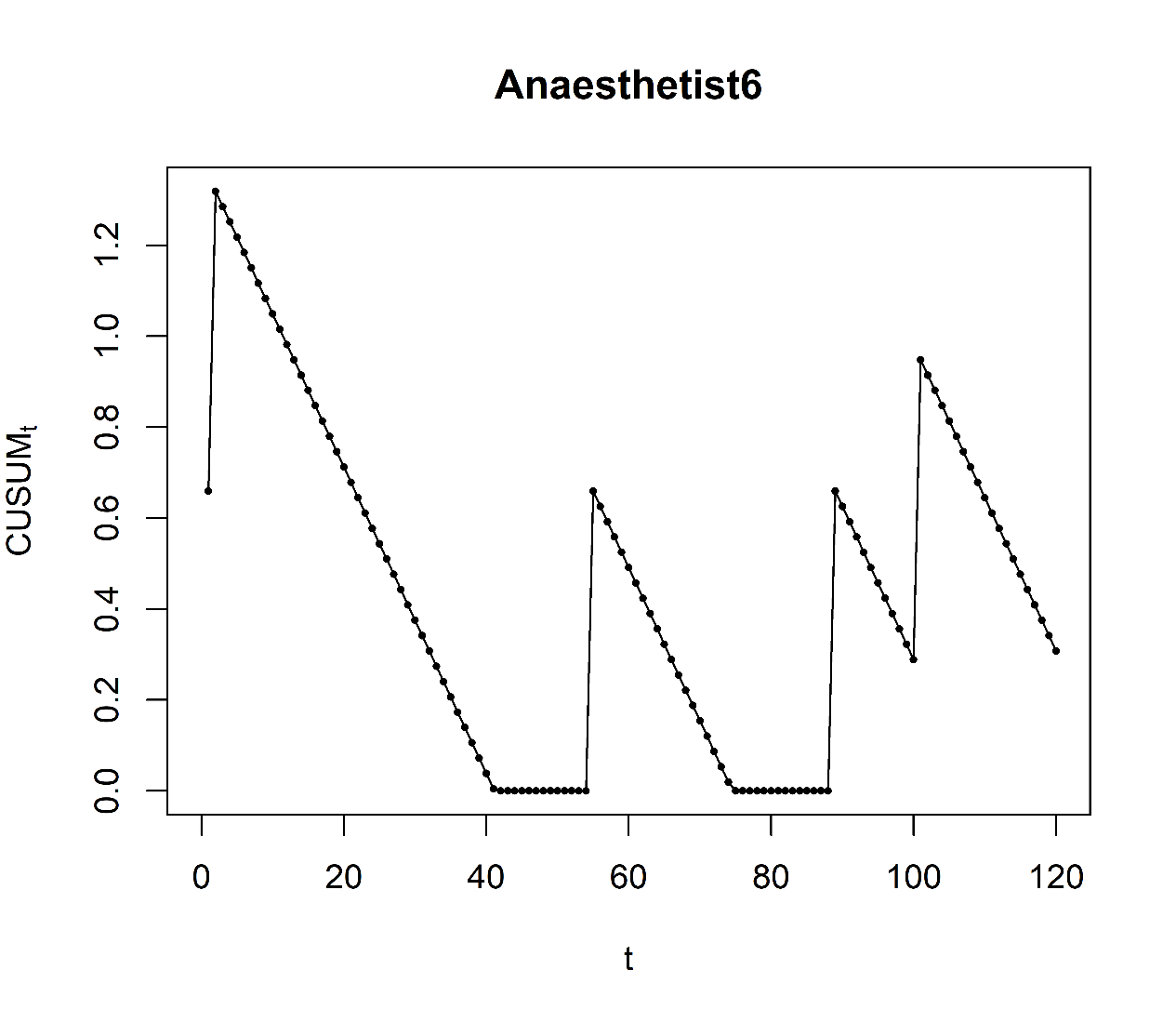

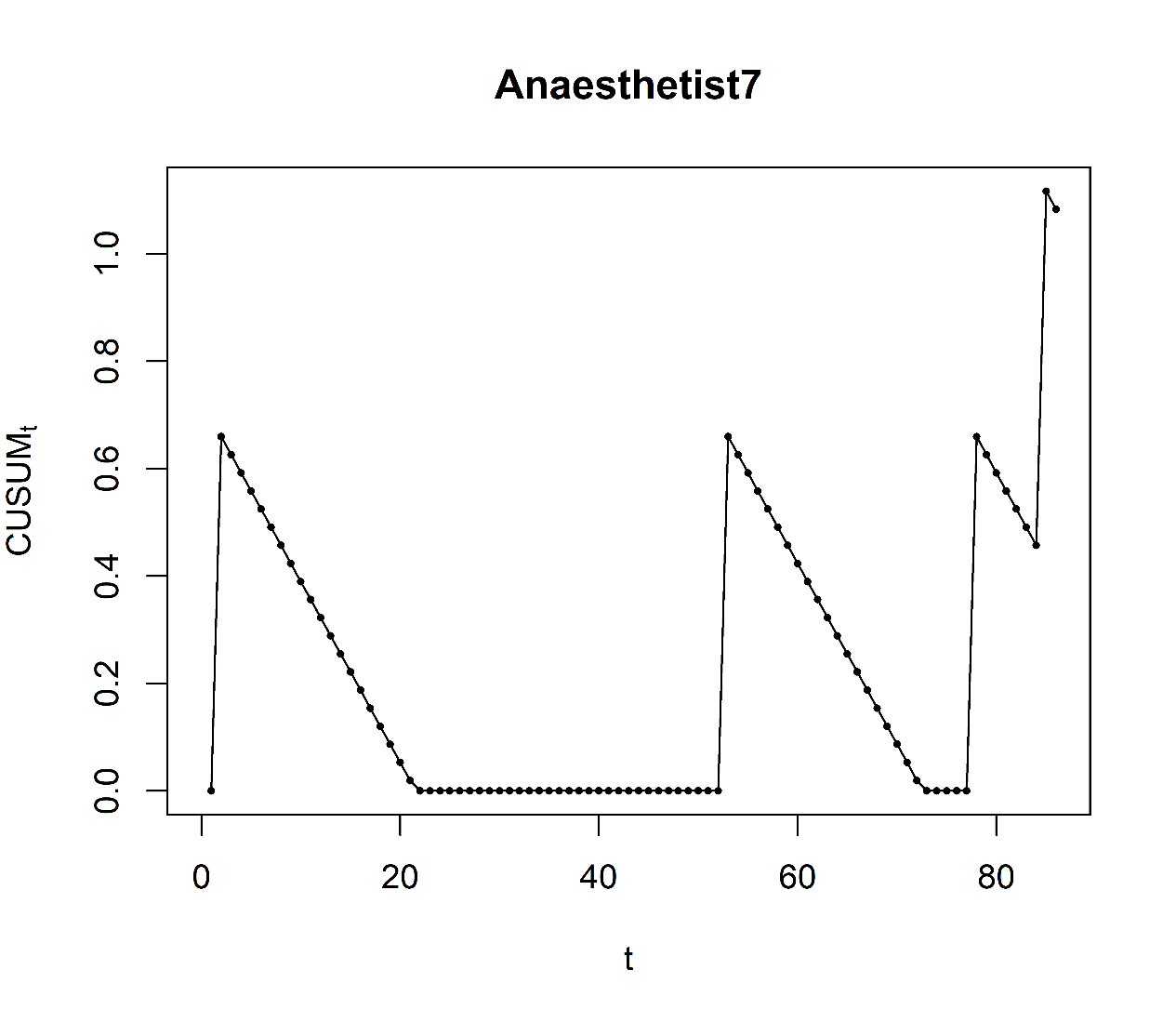

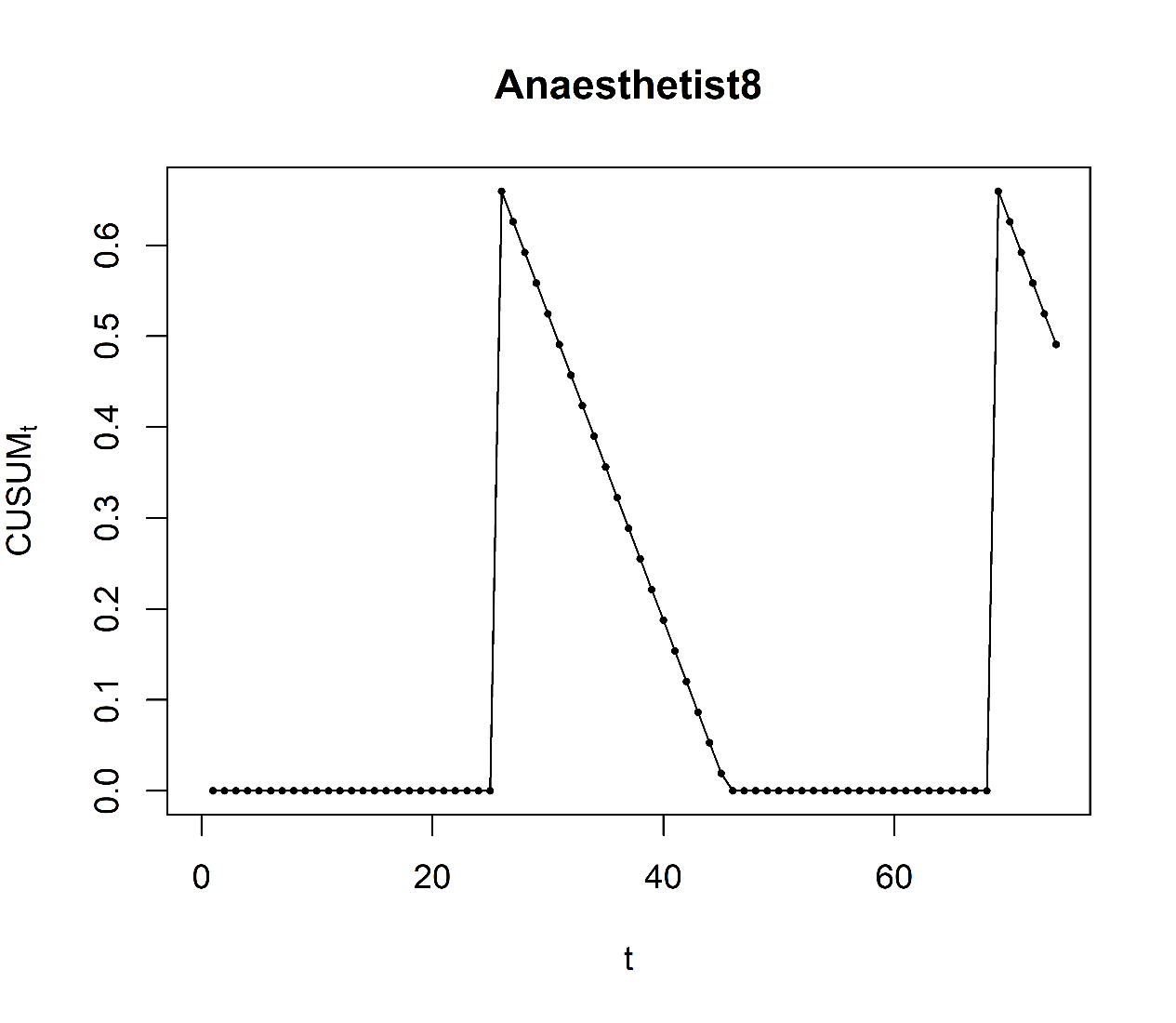

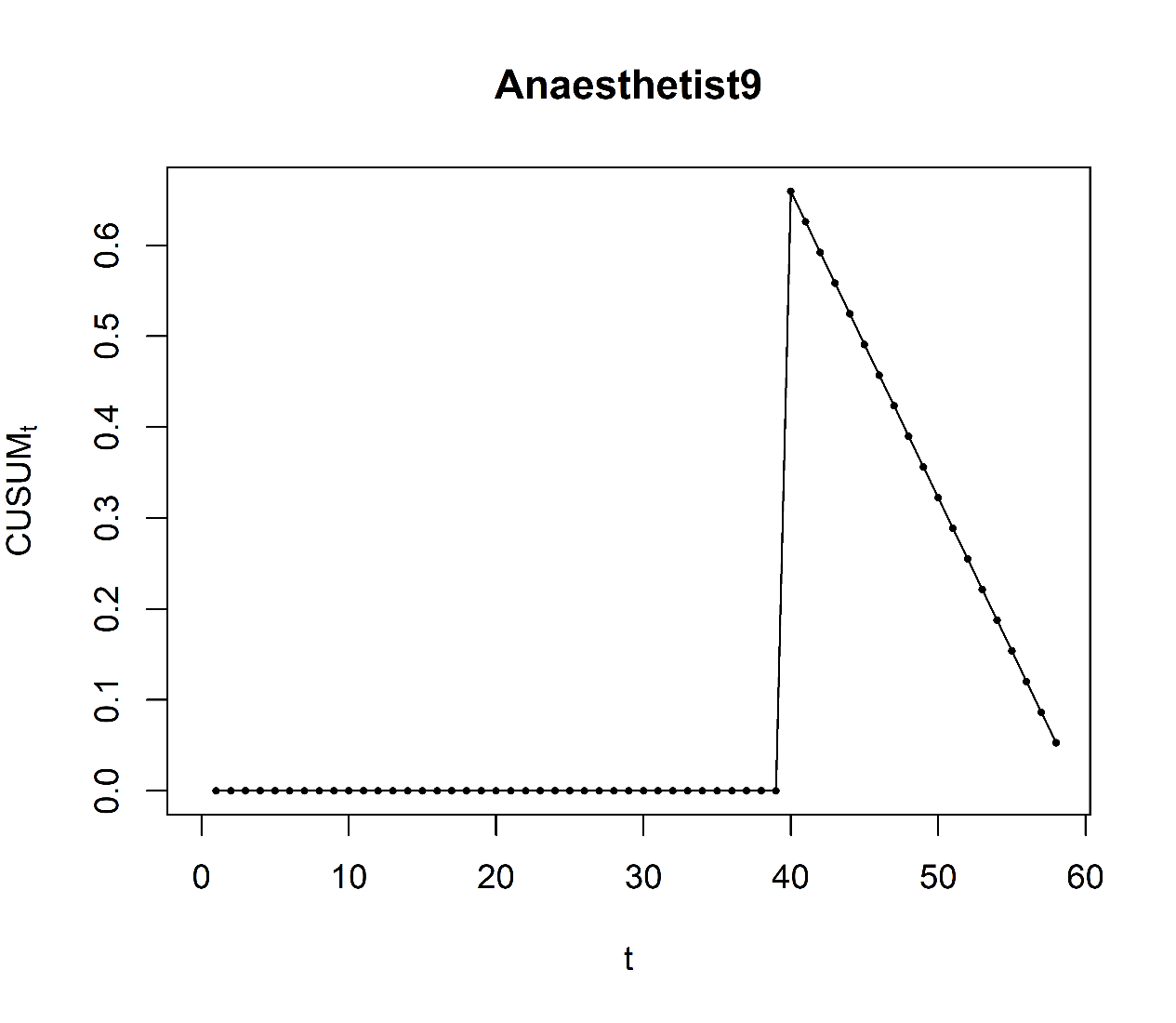

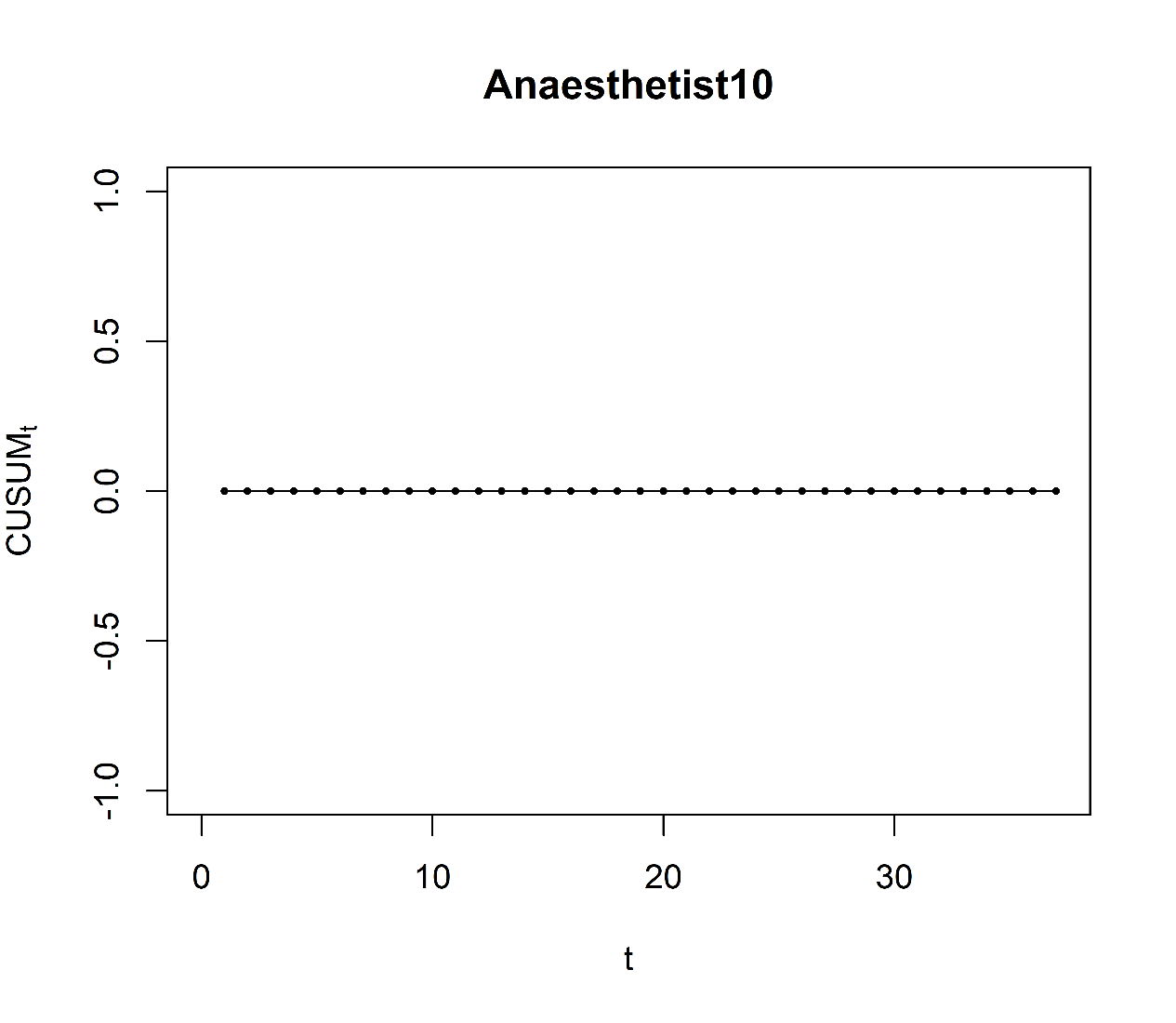

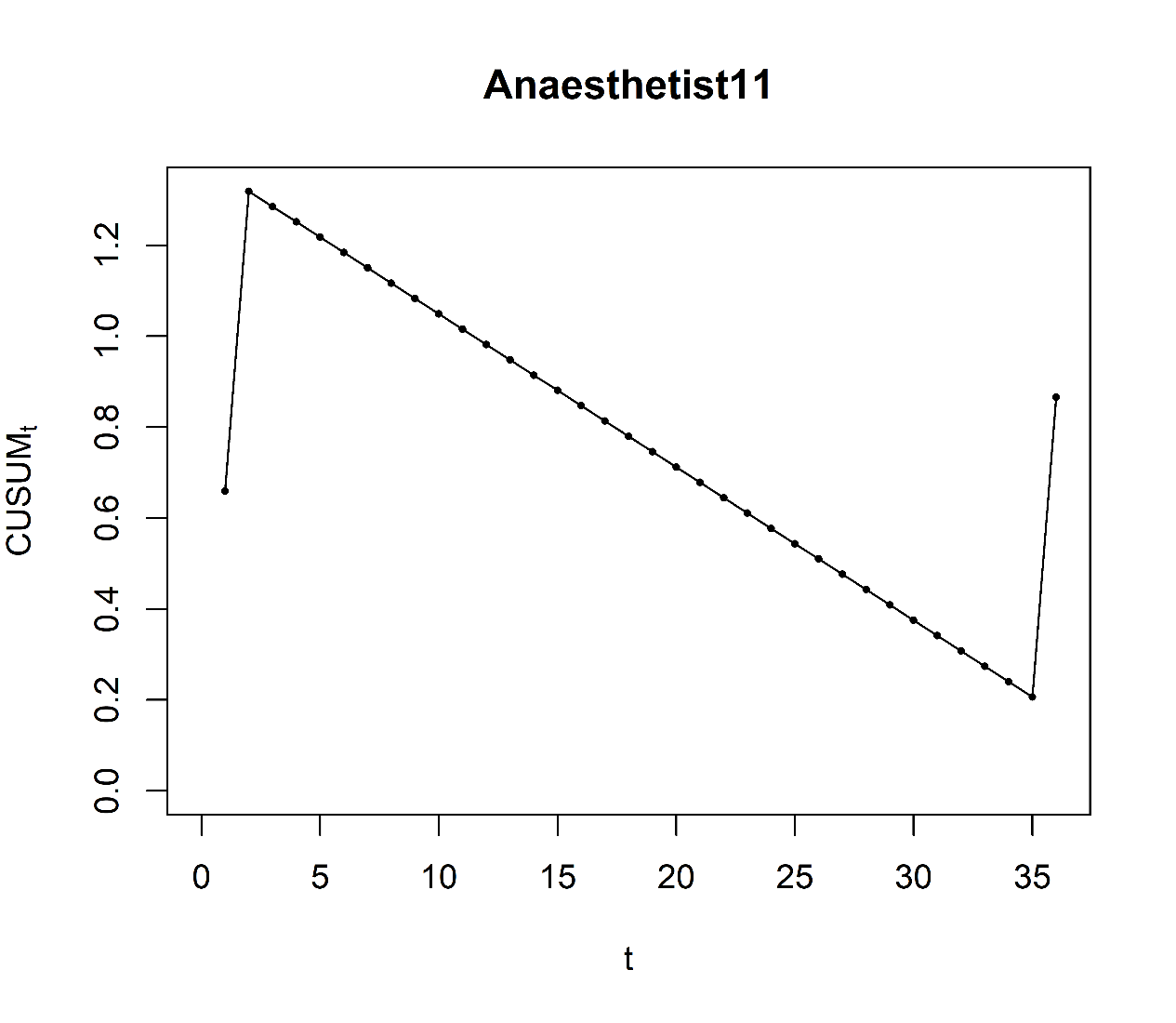

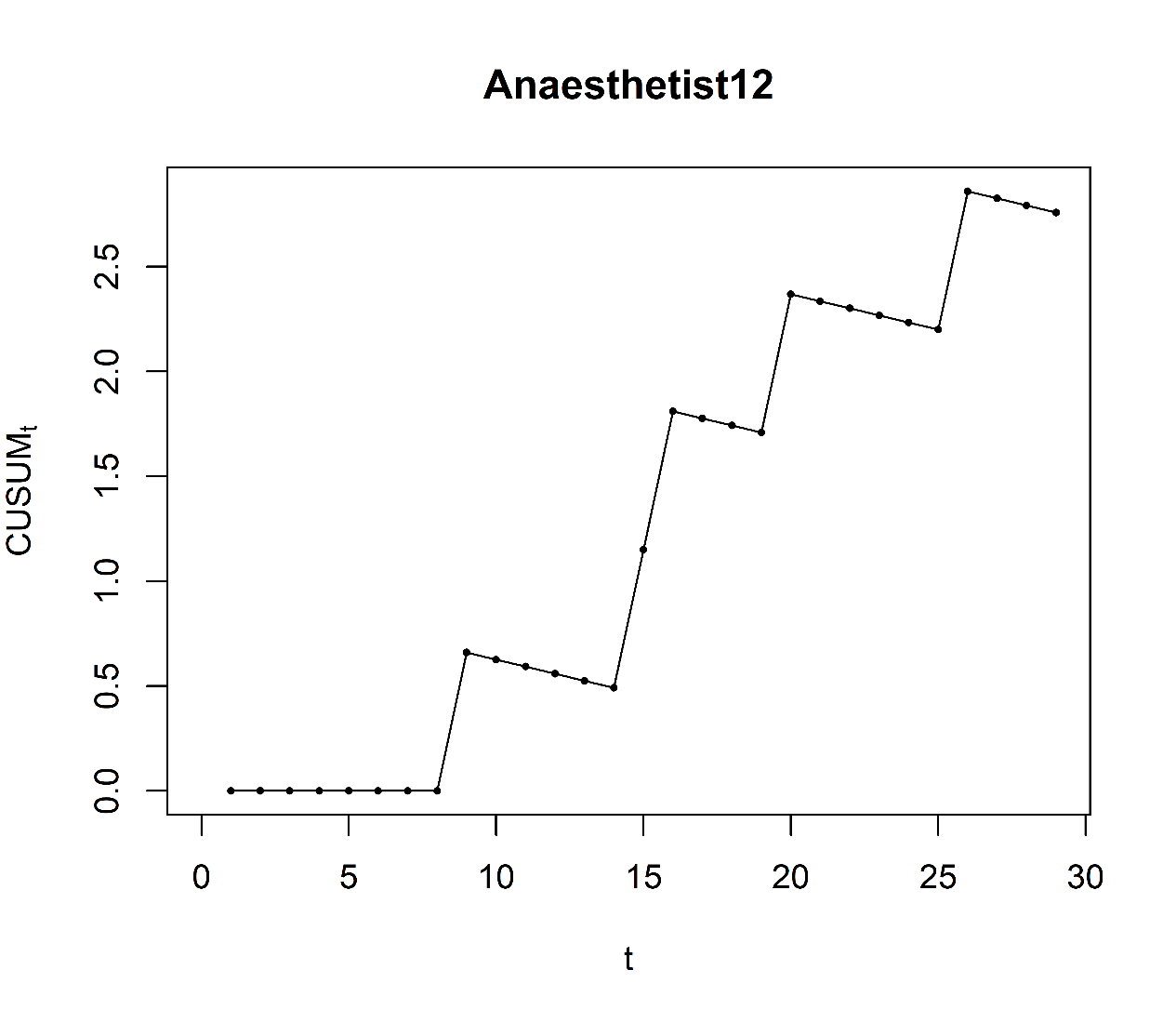

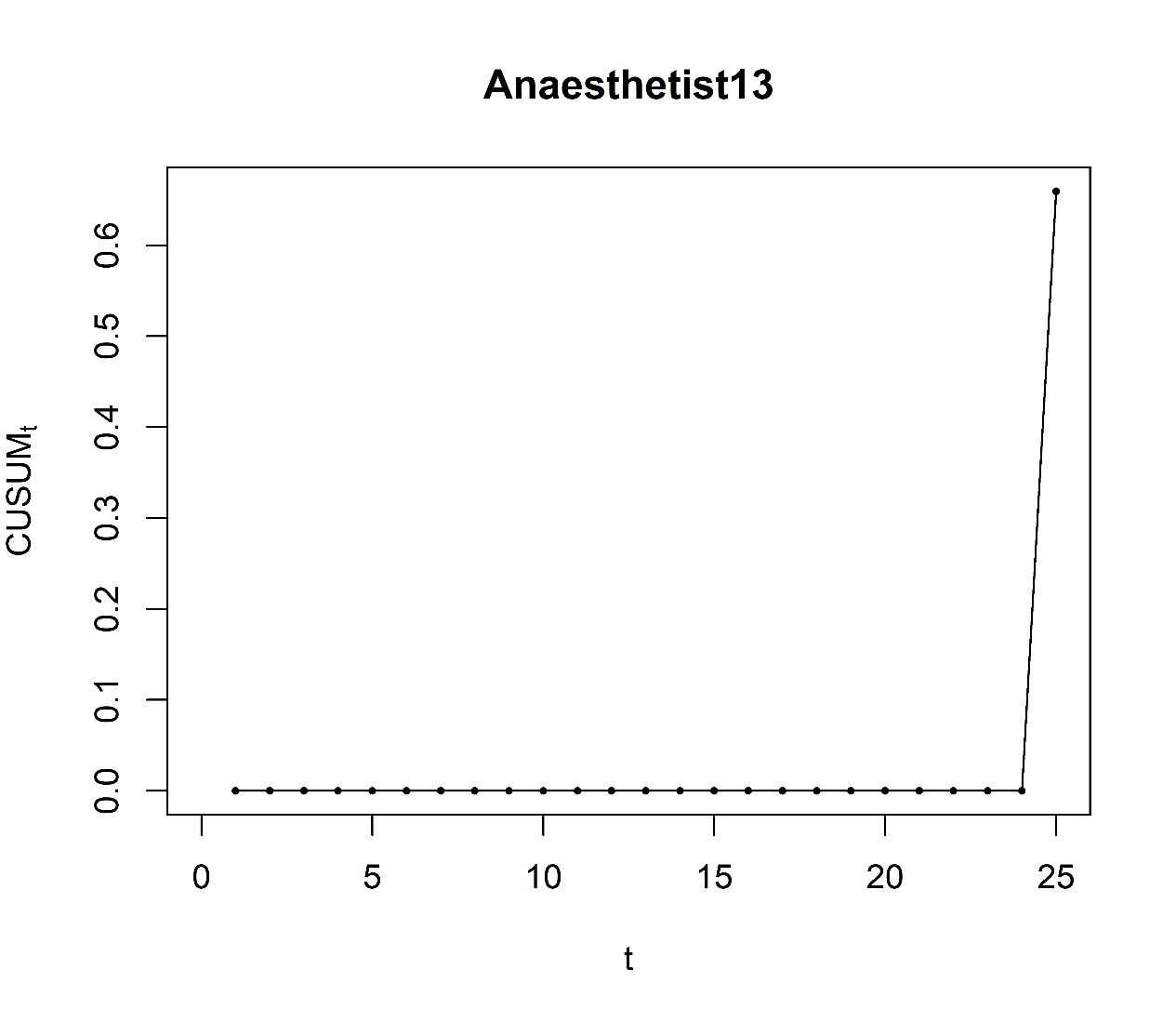

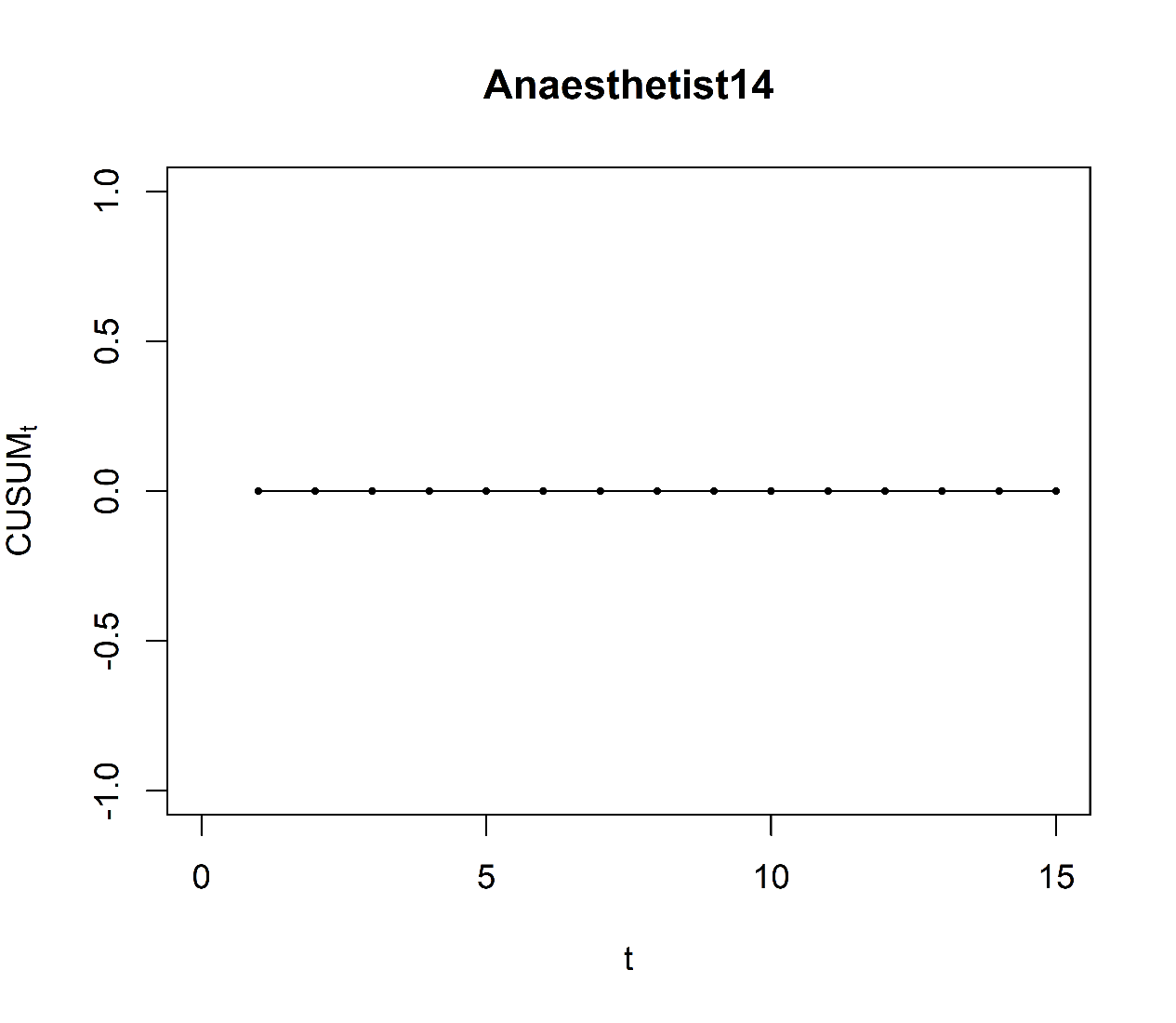

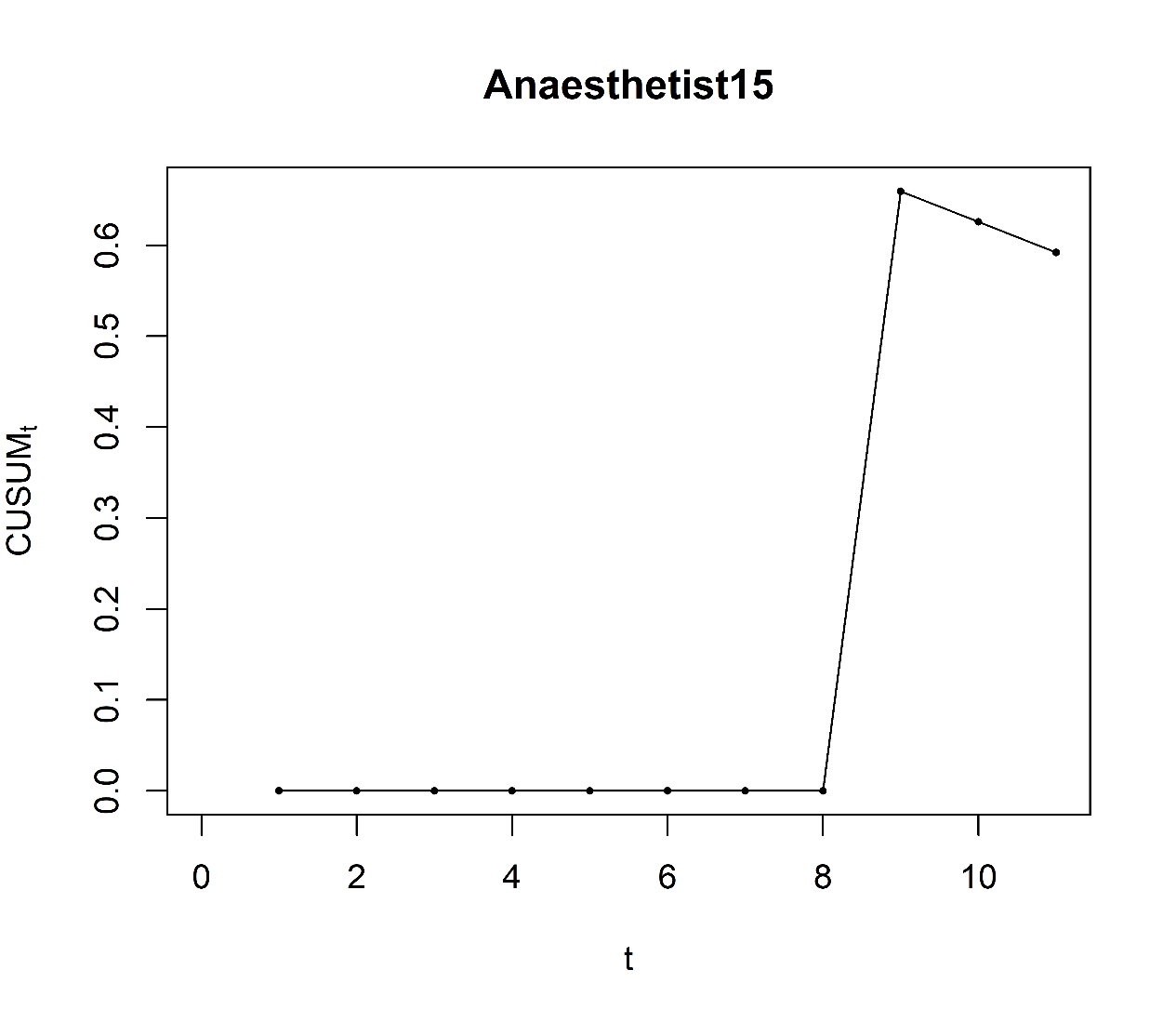

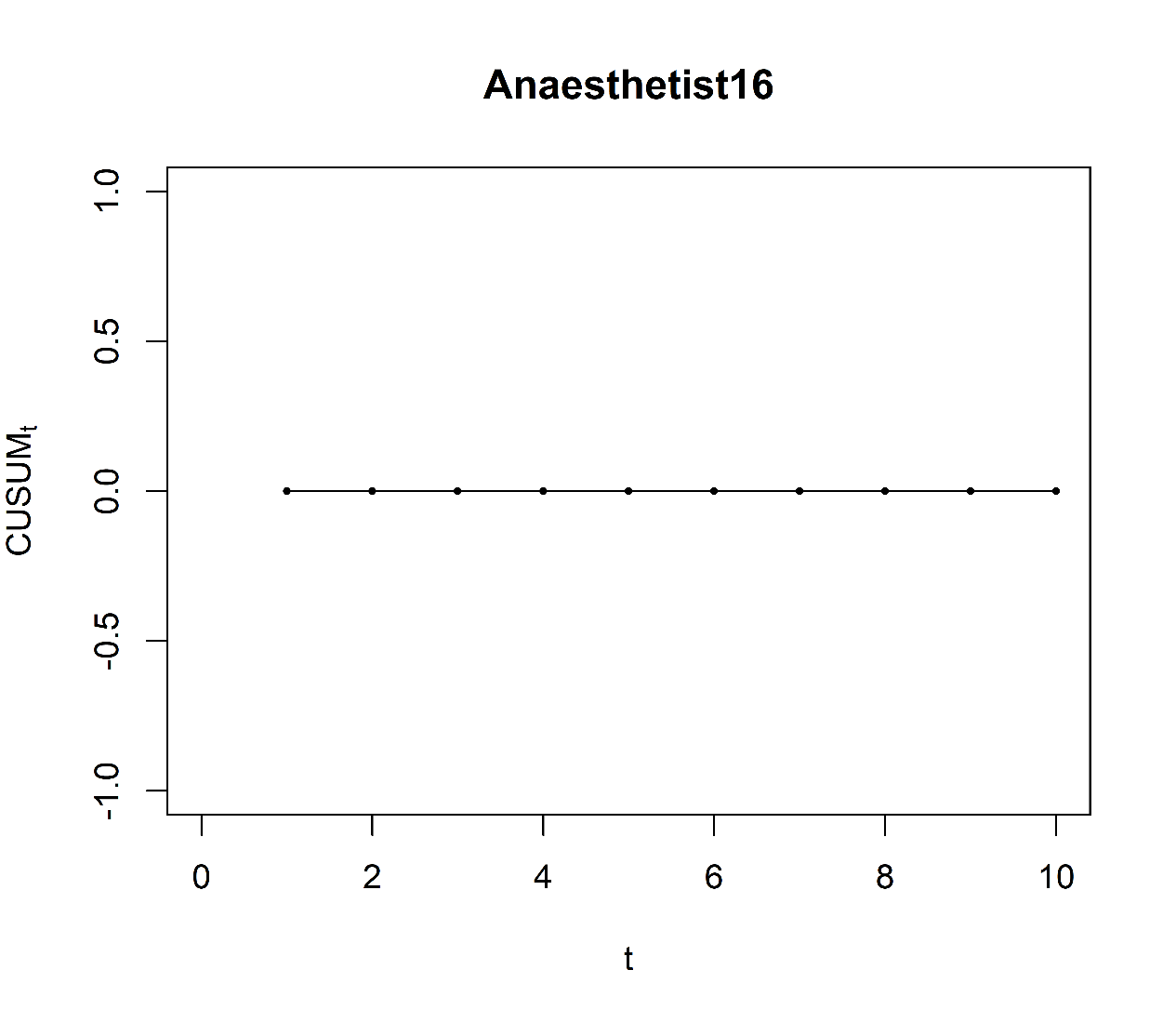

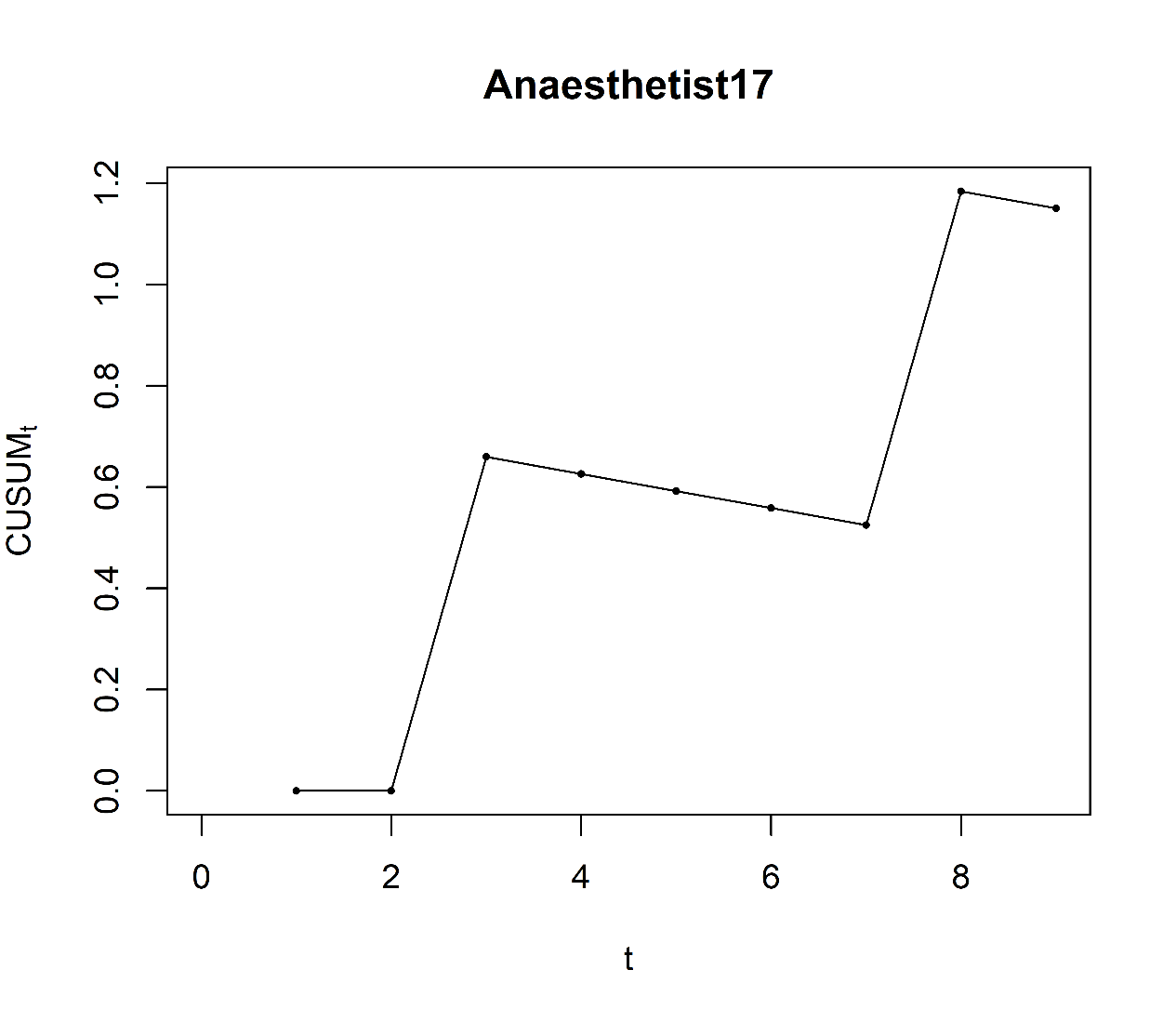

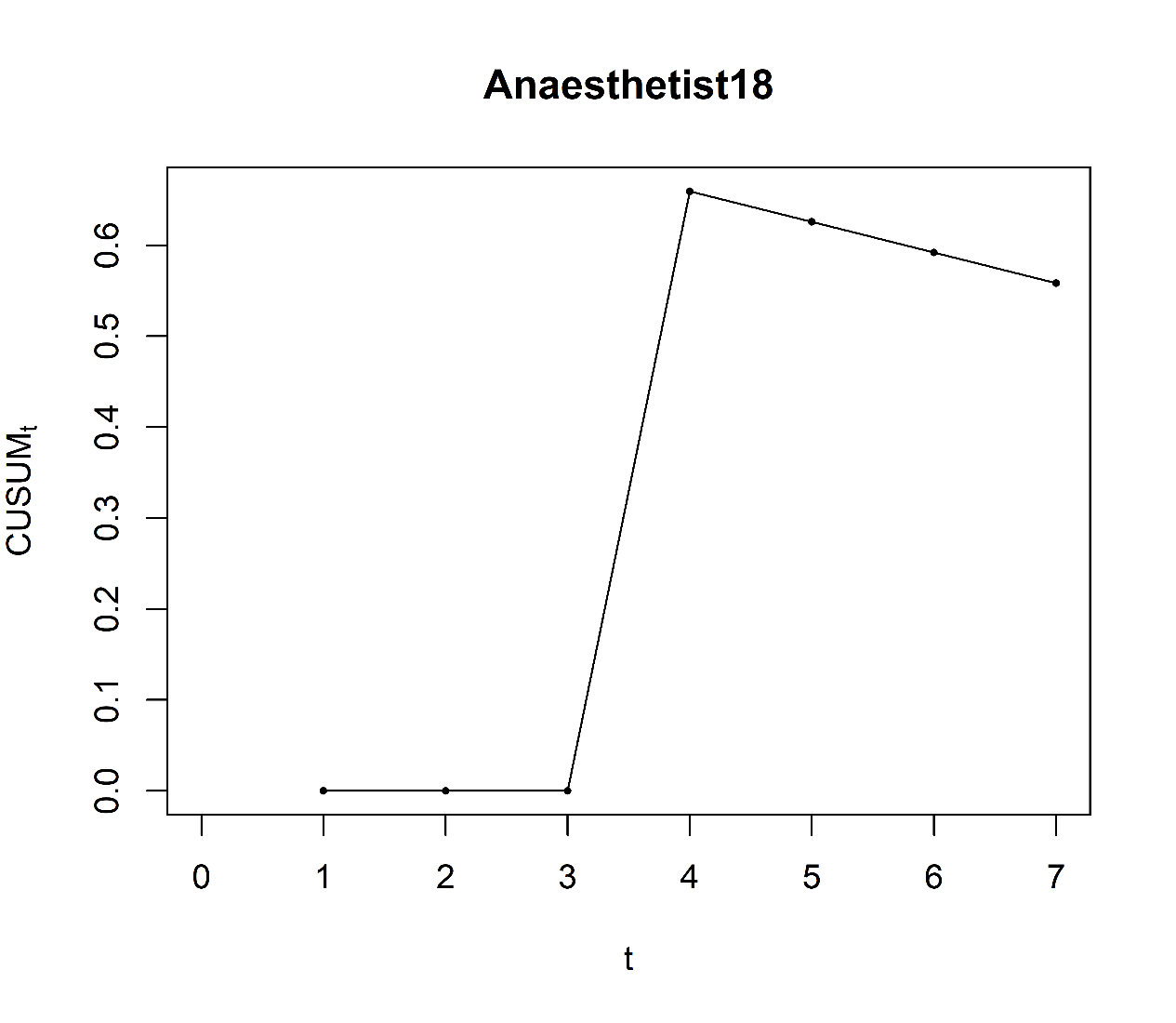

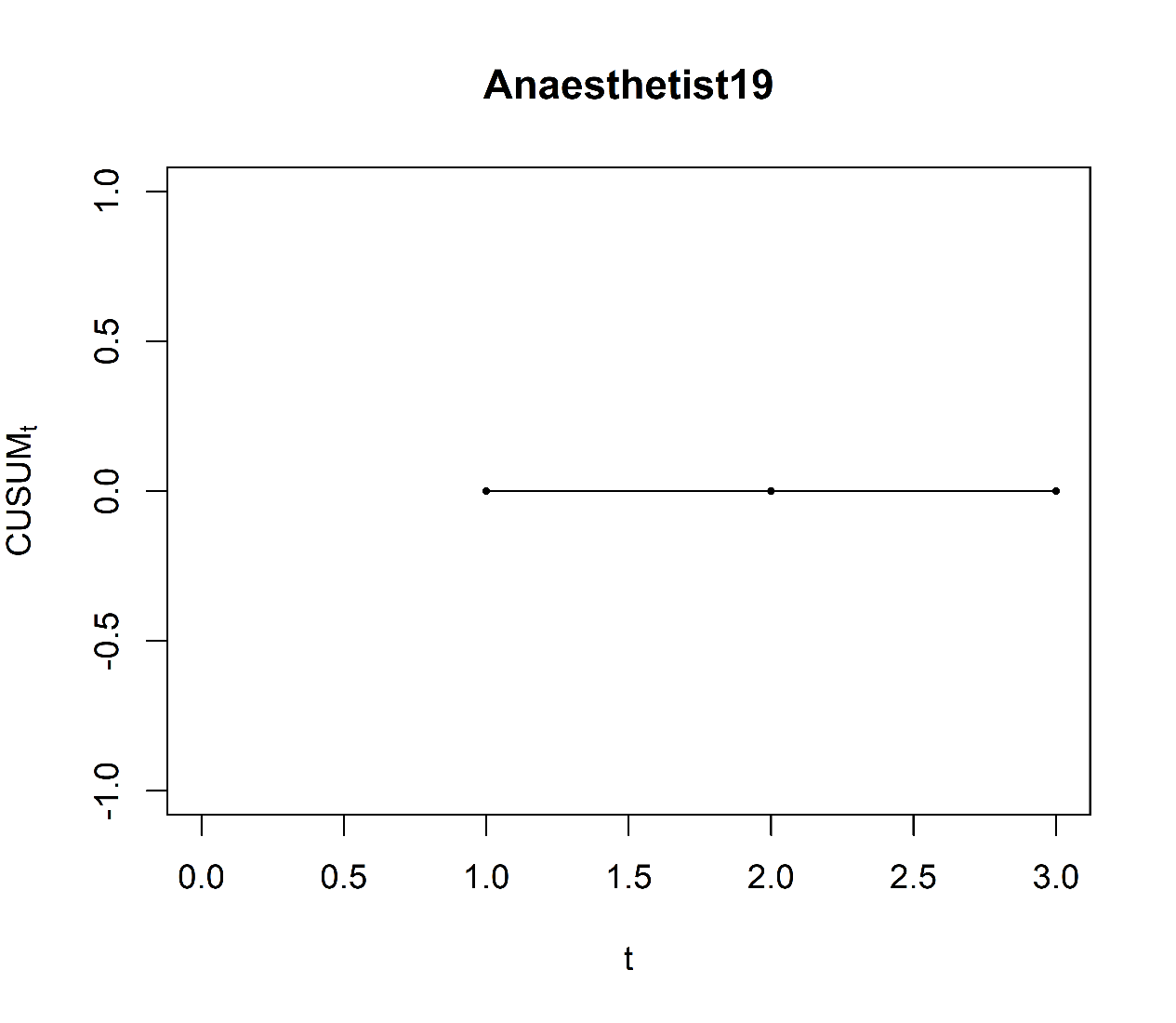

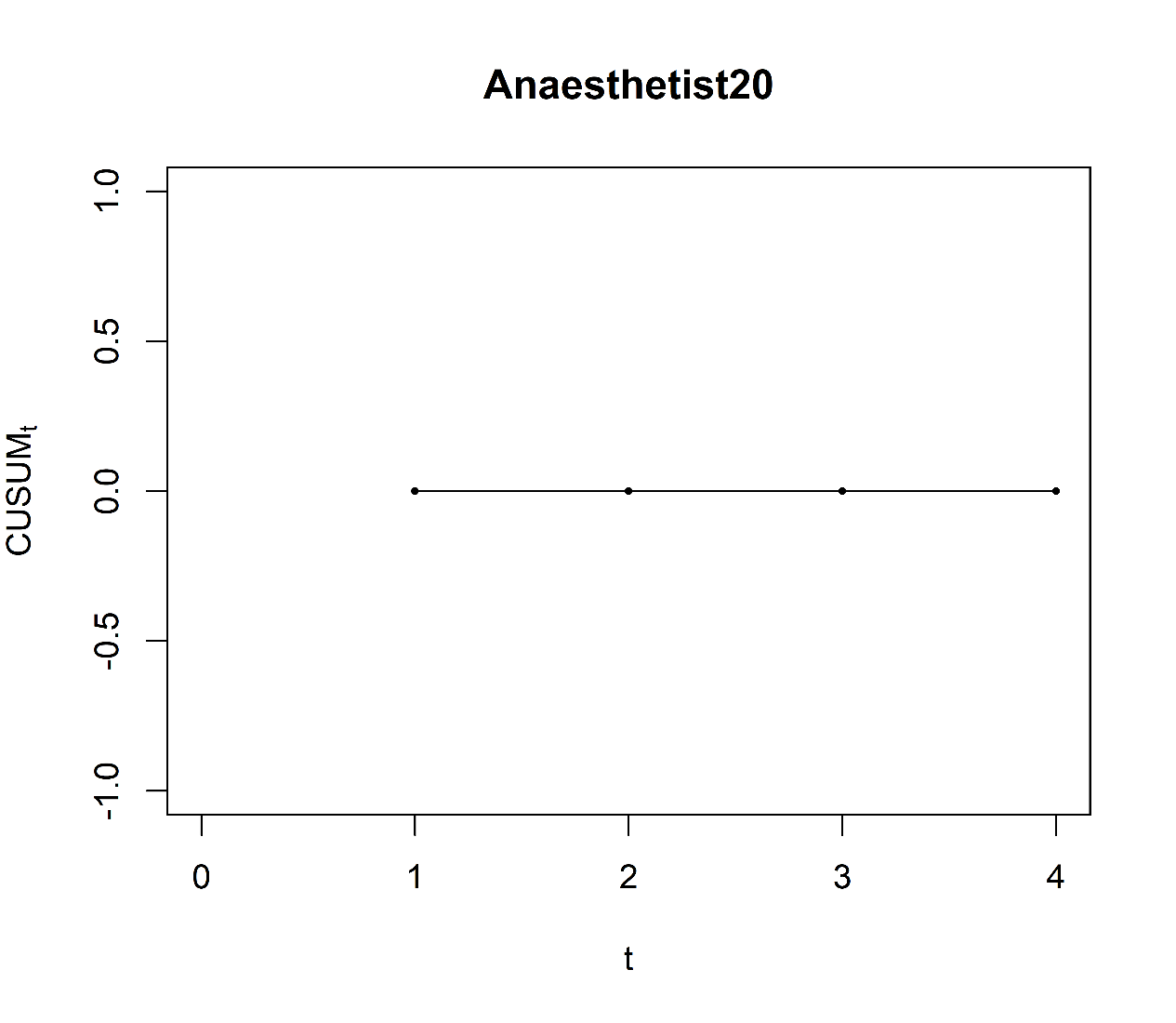

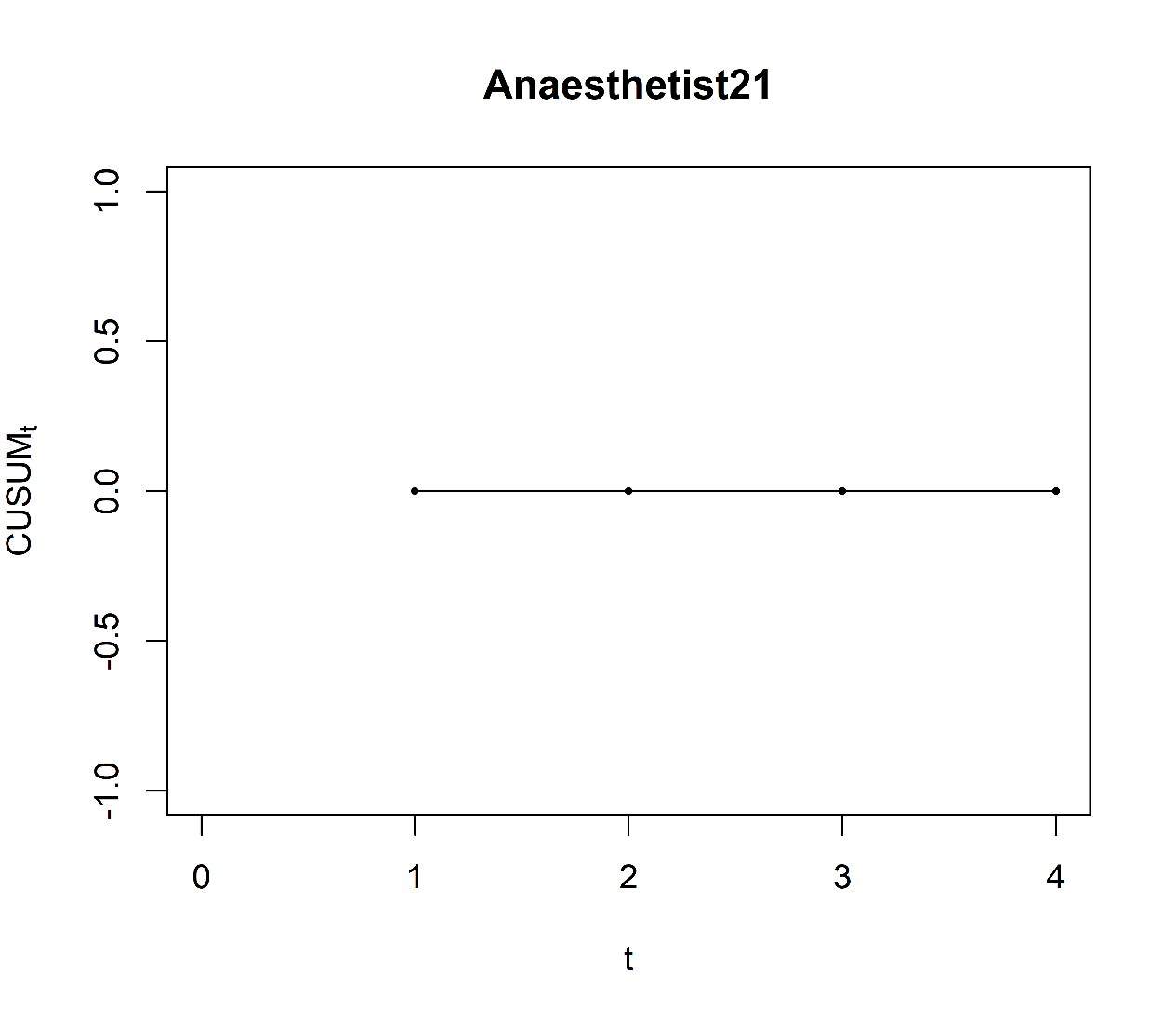

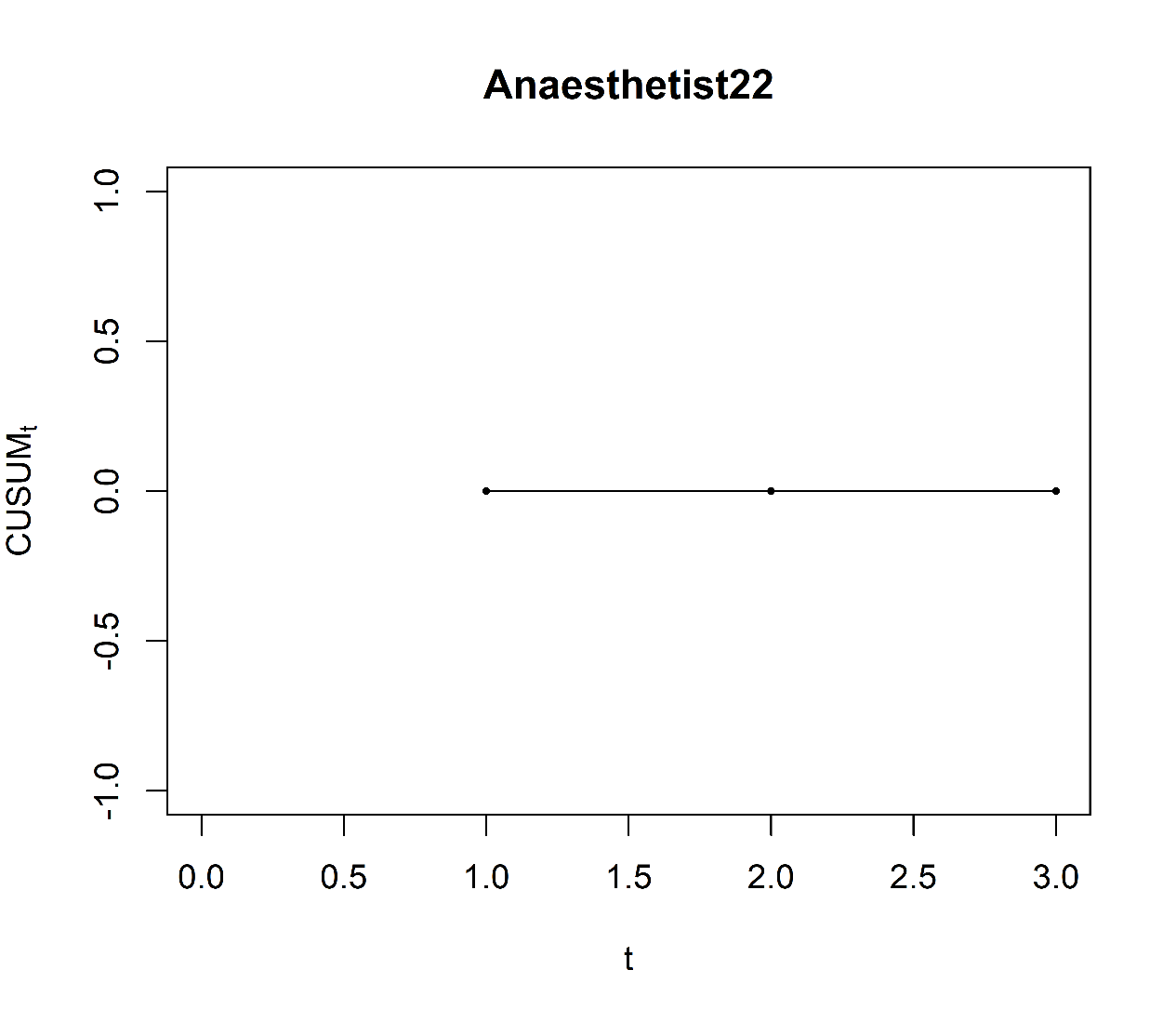

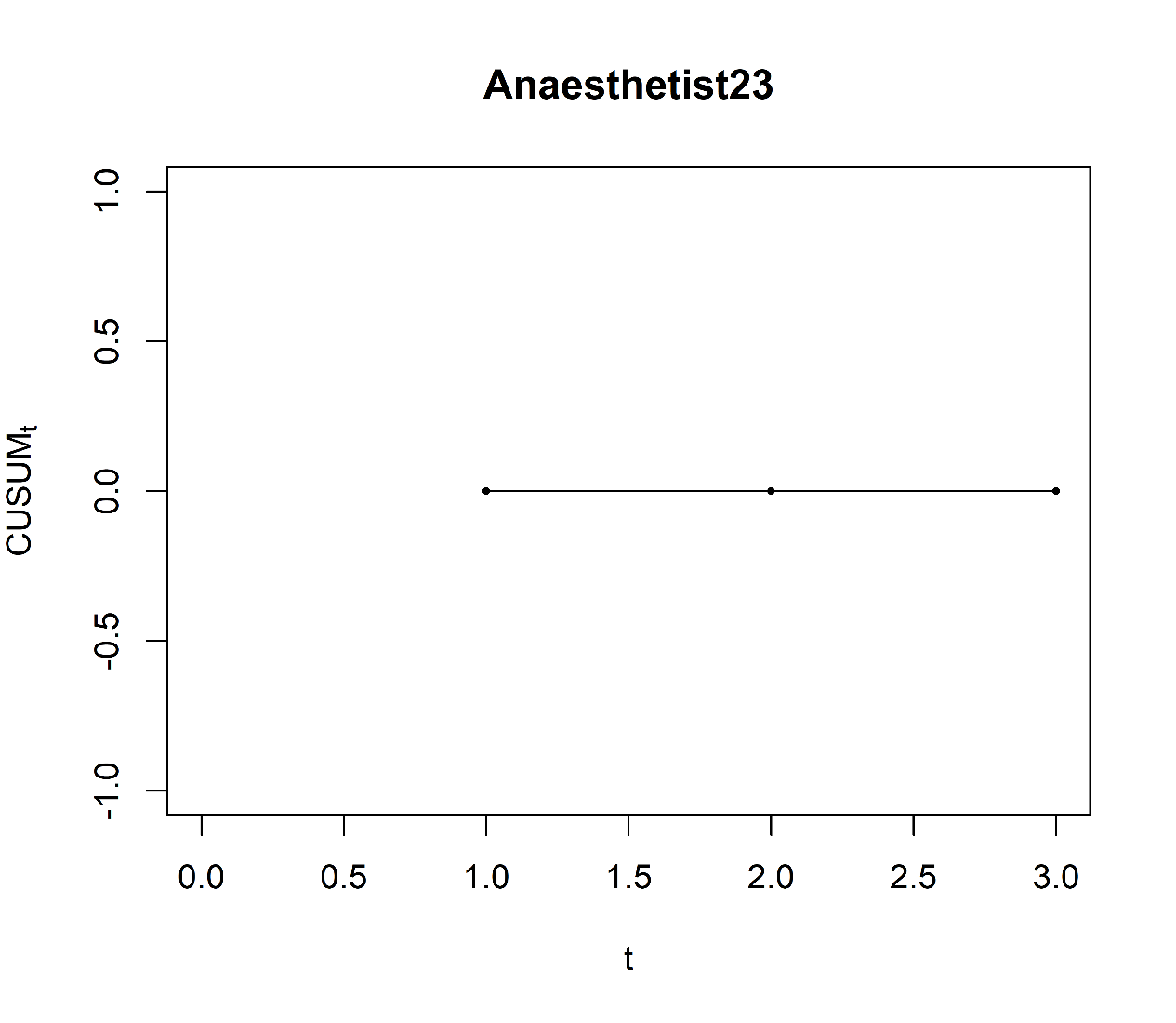

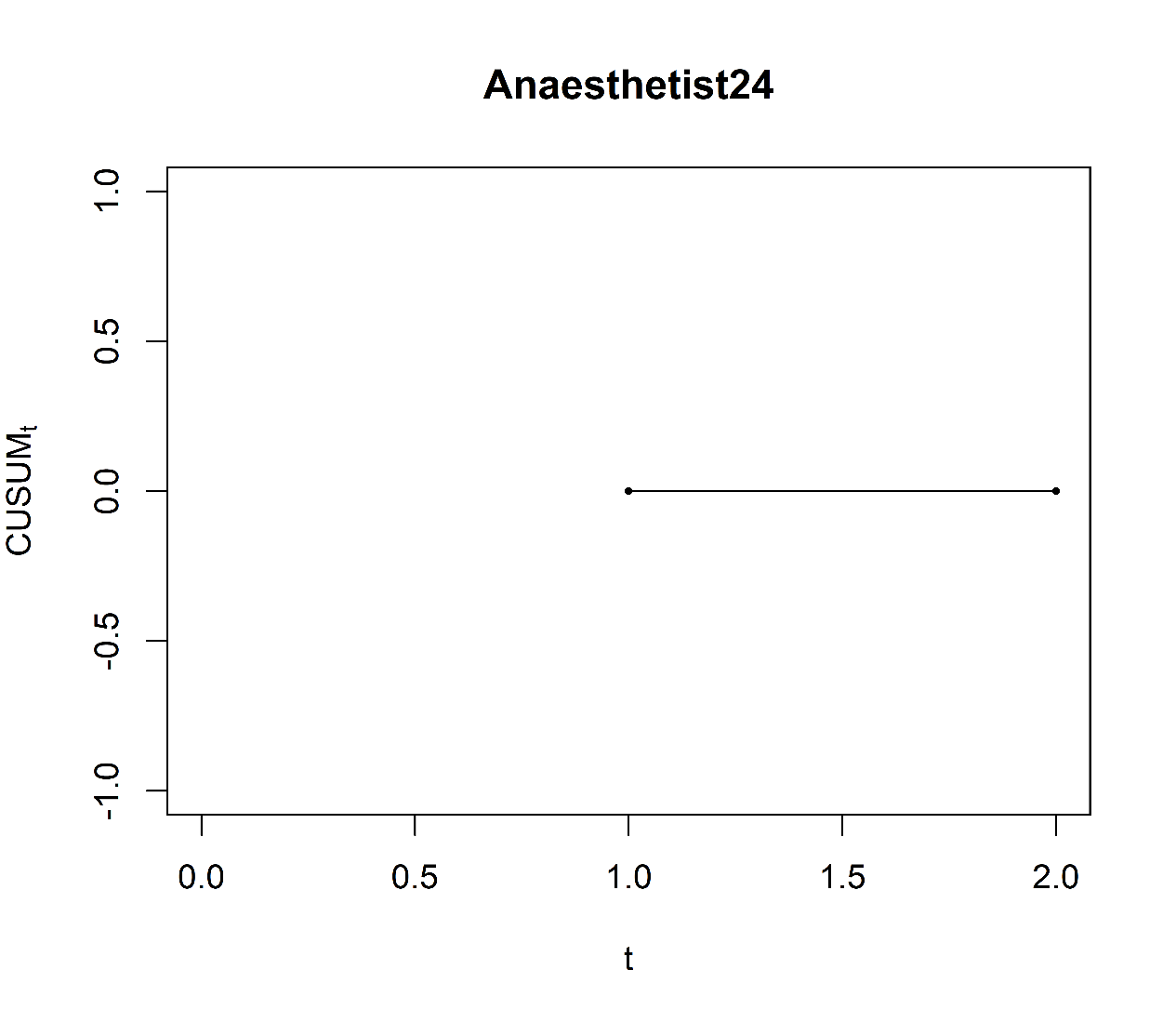

7. **Supplemental table 1. Number of conversions and procedures for individual surgeons.**

| **Supplemental table 1. Number of conversions and procedures for individual surgeons.** | | |
| --- | --- | --- |
|  | Number of conversions, n (%) | Number of procedures, n (%) |
| Surgeon1 | 32 (3.5) | 914 (33.3) |
| Surgeon2 | 25 (5.1) | 487 (17.8) |
| Surgeon3 | 6 (1.3) | 448 (16.3) |
| Surgeon4 | 18 (4) | 445 (16.2) |
| Surgeon5 | 3 (2.1) | 142 (5.2) |
| Surgeon6 | 6 (4.5) | 134 (4.9) |
| Surgeon7 | 4 (4.3) | 92 (3.4) |
| Surgeon8 | 0 | 45 (1.6) |
| Surgeon9 | 0 | 25 (0.9) |
| Surgeon10 | 0 | 8 (0.3) |
| Surgeon11 | 0 | 1 (0.03) |
| Surgeon12 | 0 | 1 (0.03) |
| Total | 94 (100) | 2742 (100) |

1. **Supplemental table 2. Number of conversions and procedures for individual anesthetists.**

| **Supplemental table 2. Number of conversions and procedures for individual anesthetists.** | | |
| --- | --- | --- |
|  | Number of conversions, n (%) | Number of procedures, n (%) |
| Anaesthetist 1 | 9 (1.5) | 613 (22.4) |
| Anaesthetist 2 | 17 (3) | 561 (20.5) |
| Anaesthetist 3 | 22 (4.6) | 481 (17.5) |
| Anaesthetist 4 | 7 (2.5) | 280 (10.2) |
| Anaesthetist 5 | 14 (5.2) | 271 (9.9) |
| Anaesthetist 6 | 5 (4.2) | 120 (4.4) |
| Anaesthetist 7 | 4 (4.7) | 86 (3.1) |
| Anaesthetist 8 | 2 (2.7) | 74 (2.7) |
| Anaesthetist 9 | 1 (1.7) | 58 (2.1) |
| Anaesthetist 10 | 0 (0) | 37 (1.3) |
| Anaesthetist 11 | 3 (8.3) | 36 (1.3) |
| Anaesthetist 12 | 5 (17.2) | 29 (1.1) |
| Anaesthetist 13 | 1 (4) | 25 (0.9) |
| Anaesthetist 14 | 0 (0) | 15 (0.5) |
| Anaesthetist 15 | 1 (9.1) | 11 (0.4) |
| Anaesthetist 16 | 0 (0) | 10 (0.4) |
| Anaesthetist 17 | 2 (22.2) | 9 (0.3) |
| Anaesthetist 18 | 1 (14.3) | 7 (0.3) |
| Anaesthetist 19 | 0 (0) | 3 (0.1) |
| Anaesthetist 20 | 0 (0) | 4 (0.1) |
| Anaesthetist 21 | 0 (0) | 4 (0.1) |
| Anaesthetist 22 | 0 (0) | 3 (0.1) |
| Anaesthetist 23 | 0 (0) | 3 (0.1) |
| Anaesthetist 24 | 0 (0) | 2 (0.1) |
| Total | 94 (100) | 2742 (100) |
